# Cost-effectiveness of fluocinolone acetonide implant (ILUVIEN®) in UK patients with chronic diabetic macular oedema considered insufficiently responsive to available therapies

**DOI:** 10.1186/s12913-018-3804-4

**Published:** 2019-01-09

**Authors:** Michal Pochopien, Annette Beiderbeck, Phil McEwan, Richard Zur, Mondher Toumi, Samuel Aballéa

**Affiliations:** 1 Creativ-Ceutical, ul. Przemysłowa 12, 30-701 Krakow, Poland; 2 Alimera Sciences Ophthalmologie GmbH, Cicerostraße 21, Handelsregister Amtsgericht Berlin, (Charlottenburg) HRB 165580 B, 10709 Berlin, Germany; 3 Health Economics and Outcomes Research Ltd, 9 Oak Tree Court, Mulberry Drive, Cardiff Gate Business Park, Cardiff, CF23 8RS UK; 4 Creativ-Ceutical, The Monadnock Building, Suite 73553 W Jackson, blvd Chicago, Chicago, IL 60604 USA; 5 Creativ-Ceutical, 215, rue du Faubourg St Honoré, 75008 Paris, France; 6 Aix-Marseille University, Jardin du Pharo, 58 Boulevard Charles Livon, 13007 Marseille, France

**Keywords:** Fluocinolone acetonide implant, Diabetic macular oedema, Cost-effectiveness, Treatment cost, Incremental cost-effectiveness ratio

## Abstract

**Background:**

Diabetic macular oedema (DMO) may lead to visual loss and blindness. Several pharmacological treatments are available on the National Health Service (NHS) to United Kingdom patients affected by this condition, including intravitreal vascular endothelial growth factor inhibitors (anti-VEGFs) and two types of intravitreal steroid implants, releasing dexamethasone or fluocinolone acetonide (FAc). This study aimed to assess the value for money (cost-effectiveness) of the FAc 0.2 μg/day implant (ILUVIEN®) in patients with chronic DMO considered insufficiently responsive to other therapies.

**Methods:**

We developed a Markov model with a 15-year time horizon to estimate the impact of changes in best-corrected visual acuity in DMO patients on costs and quality-adjusted life years. The model considered both eyes, designated as the “study eye”, defined at model entry as phakic with an ongoing cataract formation or pseudophakic, and the “fellow eye”. The model compared the FAc 0.2 μg/day implant with a 700 μg dexamethasone implant (pseudophakic patients only) or with usual care, defined as a mixture of laser photocoagulation and anti-VEGFs (phakic and pseudophakic patients). Costs were estimated from the perspective of the NHS and Personal Social Services; full NHS prices were used for drugs.

**Results:**

In patients who were pseudophakic at baseline, at 36 months, the FAc implant provided an additional gain of 4.01 and 3.64 Early Treatment Diabetic Retinopathy Study (ETDRS) letters compared with usual care and the dexamethasone implant, respectively. Over the 15-year time horizon, this translated into 0.185 additional quality-adjusted life years (QALYs) at an extra cost of £3066 compared with usual care, and 0.126 additional QALYs at an extra cost of £1777 compared with dexamethasone. Thus, incremental cost-effectiveness ratios (ICERs) were £16,609 and £14,070 per QALY gained vs. usual care and dexamethasone, respectively. In patients who were phakic at baseline, the FAc 0.2 μg/day implant provided an additional gain of 2.96 ETDRS letters at 36 months compared with usual care, which, over 15 years, corresponded to 0.11 additional QALYs at an extra cost of £3170, resulting in an ICER of £28,751 per QALY gained.

**Conclusion:**

The FAc 0.2 μg/day implant provided good value for money compared with other established treatments, especially in pseudophakic patients.

**Electronic supplementary material:**

The online version of this article (10.1186/s12913-018-3804-4) contains supplementary material, which is available to authorized users.

## Background

Diabetes is a common and chronic health condition with a global prevalence of 8.5% in the adult population [1]. In the UK, almost 3.6 million people are living with this disease [2]. Those suffering from diabetes may experience long-term complications impacting various parts of the body, including kidney failure, cardiovascular events, lower extremity amputations and loss of vision [1]. The latter is related to diabetic retinopathy, which affects approximately 35% of diabetics, threatening the vision of 7% [1]. However, certain populations, such as those with longer duration of diabetes or type 1 disease, are more frequently affected [1].

Diabetic macular oedema (DMO) is a complication of diabetic retinopathy characterised by increased vascular permeability, where leakage of fluid from blood vessels causes swelling of the retina, which may lead to visual loss and – eventually – blindness [3, 4]. DMO is the most common cause of moderate visual loss from diabetic retinopathy [4], with an estimated age-standardised prevalence of 6.81% amongst patients with diabetes [5]. However, DMO can be far more common in certain populations, with age-standardised prevalence approaching 15% in patients with type 1 diabetes and 20% in those who have had diabetes for ≥20 years [5]. Recent studies from the UK reported an estimated DMO prevalence of 7.12% amongst diabetics in England (with the condition affecting both eyes in 2.34% of diabetes patients) [6, 7].

A number of drugs used to treat DMO are available to UK patients on the National Health Service (NHS), following a positive recommendation from the National Institute for Health and Care Excellence (NICE). These include the anti-VEGFs aflibercept and ranibizumab, recommended in patients whose eye has a central retinal thickness of ≥400 μm at the start of treatment, and two steroid implants – a short-acting [8] one that uses dexamethasone, and a long-acting [9] fluocinolone acetonide (FAc) implant (ILUVIEN®) [10, 11]. The main mechanism of action is the induction of phospholipase A inhibitory proteins, collectively called lipocortins. These proteins control the biosynthesis of potent mediators of inflammation such as prostaglandins and leukotrienes by inhibiting the release of the common precursor arachidonic acid. Arachidonic acid is released from membrane phospholipids by phospholipase A2. Corticosteroids have also been shown to reduce levels of vascular endothelial growth factor, a protein which increases vascular permeability and causes oedema.

There is a substantial body of evidence supporting the use of the FAc implant in DMO. Although, it is licensed for use in DMO patients regardless of their lens status [9]. NICE recommended restricting the use of the FAc implant on the NHS to pseudophakic eyes only. It is an eye in which the natural lens is replaced with an intraocular lens, since cost-effectiveness results in the full DMO population were associated with substantial uncertainty. However, the analysis in the full population was confounded by the formation of cataracts in phakic eyes, and the resulting uncertainty in BCVA gain extrapolations. Furthermore, recent evidence shows that the FAc 0.2 μg/day implant provides cost savings to the NHS versus continued ranibizumab therapy, irrespective of lens status [12]. This is of particular importance considering that in UK clinical practice many patients who insufficiently respond to anti-VEGFs continue to receive them. A recent post-hoc analysis of the Diabetic Retinopathy Clinical Research (DRCR) Network’s Protocol I study showed that approximately 40% of patients treated with ranibizumab responded to it insufficiently [13]. Consequently, patients are potentially exposed to burden some intravitreal injections, despite not experiencing sufficient benefits while the NHS incurs unnecessary costs of an intervention lacking real-world effectiveness. Thus, ensuring adequate treatment options are available to patients who do not sufficiently respond to anti-VEGFs is crucial, both from the clinical and health economics perspectives.

The objective of this study was therefore to assess the cost-effectiveness of the FAc 0.2 μg/day implant in patients insufficiently responsive to other therapies, compared with: 1) usual care, consisting of laser photocoagulation and anti-VEGFs, and 2) dexamethasone implant.

## Methods

### Study population

The modelled population consisted of adult patients with chronic DMO in at least one eye, which the treating physician deemed insufficiently responsive to usual care. Two sub-populations were evaluated: 1) pseudophakic patients (i.e. patients with an intraocular lens implanted during cataract surgery), 2) phakic patients experiencing some opacities, indicating the development of a cataract in the study eye (hereafter referred to as “phakic patients”).

### Model structure

**Fig. 1:**
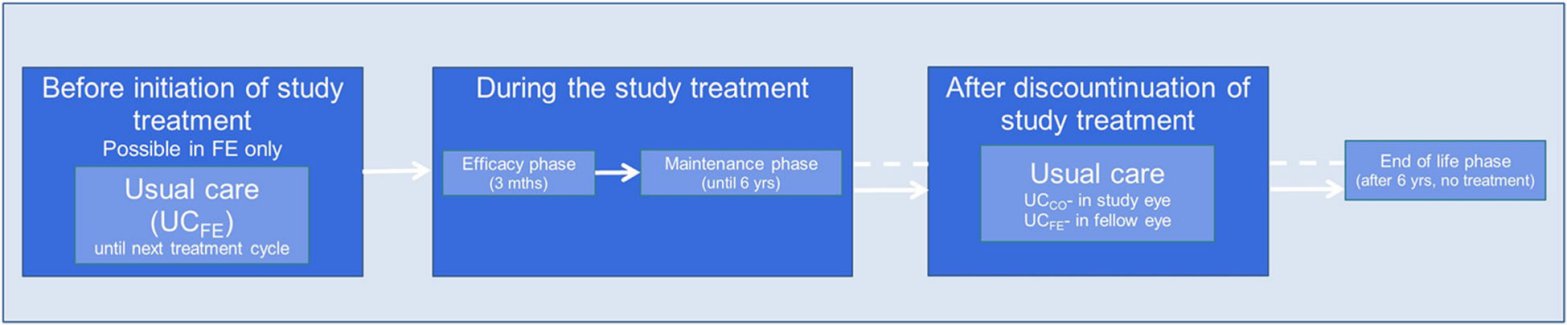
Treatment phases

**Fig. 2:**
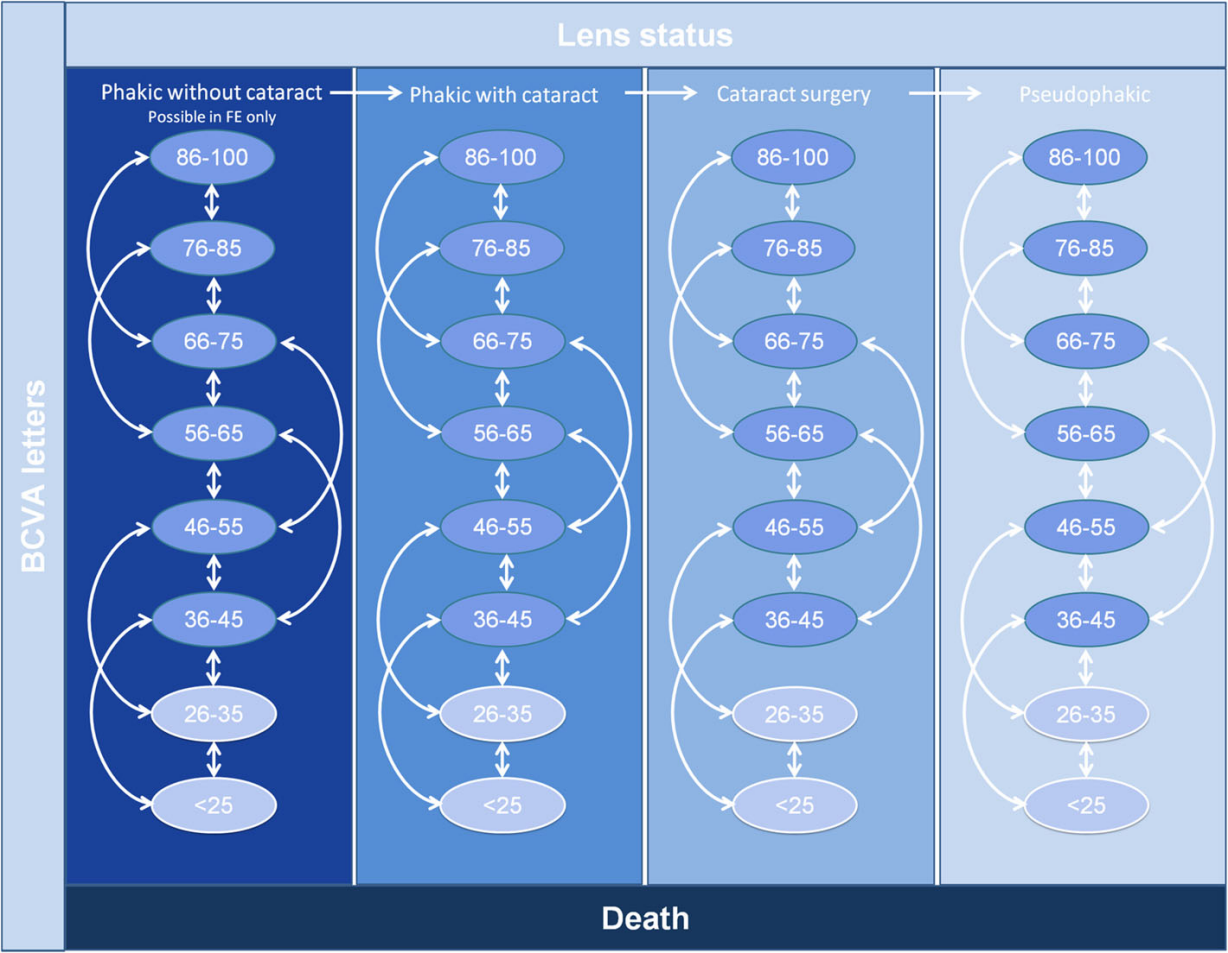
Model structure

We developed a Markov cohort model with a 15-year time horizon and 3-month cycle length that is structurally consistent with previously published models [14] to estimate the impact of changes in best-corrected visual acuity (BCVA) in DMO patients on costs and quality adjusted life years. The model considered both eyes, designated as the “study eye” (the eye in which the study treatment was initiated) and the “fellow eye”. The health states for each eye were defined according to BCVA (8 score levels) [15, 16], lens status (either eye could be phakic without cataract, phakic with cataract, phakic with cataract undergoing a cataract surgery, or pseudophakic), treatment phase (Fig. 1) and – in the fellow eye only – DMO status (patients could, but did not have to, have DMO in the fellow eye) (Fig. 2 and Table 1**)**.

**Table 1:** Key features and assumptions of the model

Treatment duration	The total treatment period could not exceed 6 years, regardless of treatment strategy used.
Treatment phases	In order to fit the BCVA evolution profile observed in clinical trials [35, 36] treatment response was modelled through three phases over time. The first (efficacy) phase, lasting 3 months, was where the active treatments had a positive impact on BCVA levels. This was followed by the maintenance phase lasting until the end of year 6, characterised by slower improvement in BCVA over time. The final end-of-life phase, starting six years from model entry, assumed a constant decline of vision over time. Of note, although the response to treatment varied with these phases, treatment doses did not.
Treatment in one or both eyes	At baseline, patients were treated for DMO in the study eye only, or in both eyes. The latter group could receive different treatments in both eyes, and if their fellow eye did not receive the study treatment, it was assumed to be treated with usual care (UC).
Treatments included in usual care	Based on the ICE-UK study, UC was defined as a mixture of laser photocoagulation, ranibizumab 0.5 mg/injection, bevacizumab 1.25 mg/injection and aflibercept 2 mg/injection. For treatment mix see Table 2.
Implant discontinuation	Patients who discontinued treatment with implants in either eye were switched to UC in that eye.
Usual care administration	We did not consider any fixed schedule of laser administration and anti-VEGF injection, but the model took into account the average number of administrations / injections per year in clinical practice (see Table 2)
Anti-VEGF efficacy	The efficacy of anti-VEGFs in the study eye was assumed to be insufficient based on the population definition. Therefore, the costs of UC included anti-VEGF costs, but the efficacy of UC was conservatively assumed to be identical to that of sham plus usual care in the FAME study. We also performed a scenario analysis where UC costs included only the costs of laser photocoagulation, and BCVA was assumed to be constant. Fellow eyes treated with UC were potentially responsive to anti-VEGFs.
Other treatment combinations	In clinical practice, patients may receive anti-VEGF injections alongside steroid implants. The costs and adverse effects of those injections were not explicitly considered in FAc and dexamethasone arms, but the numbers of anti-VEGF injections in the UC arm was reduced to avoid any bias in favour of steroids.

Anti-VEGF = Anti- Vascular Endothelial Growth Factor; BCVA = Best Corrected Visual Acuity; DMO = Diabetic Macular Oedema; FAc = fluocinolone acetonide; UC = Usual Care

### Treatment strategies

The comparators included in the model represent therapies that may be available in the UK to DMO patients based on their clinical profile, including 1) intravitreal implant containing 190 micrograms of FAc (ILUVIEN®) [9] administered every 36 months, 2) intravitreal implant containing 700 micrograms of dexamethasone [8] administered every 6 months, 3) usual care (UC), defined as a mixture of laser photocoagulation, ranibizumab 0.5 mg, bevacizumab 1.25 mg and aflibercept 2 mg, based on the ILUVIEN Clinical Evaluation-UK (ICE-UK) study **(**Additional file 1: Figure S1**)**.

### Disease progression

#### Transition probabilities for FAc implant in both eyes and usual care in the study eye

Two multinomial logistic regression models were developed using patient-level data from the FAME clinical trial [17] to derive transition probabilities for patients’ subgroups based on lens status.

For the chronic pseudophakic population, the probability of being at BCVA level j at month (t + 3) was expressed as a function of treatment, treatment phase (efficacy or maintenance phase, i.e. 0 to 3 months or after 3 months), BCVA level in previous visit, interaction between treatment and phase, and interaction between BCVA level in previous visit and phase.

$$ Prob\left({BCVA\ level}_{t+3}=j\ |\ x\right)=\frac{e^{\beta_0^j+{\beta}_1^j. Treatment+{\beta}_2^j.{BCVA\ level}_t+{\beta}_3^j. phase+{\beta}_4^j. reatment\ast phase+{\beta}_5^j. phase\ast {BCVA\ level}_t}}{1+\sum \limits_{k=1}^{J-1}{e}^{\beta_0^k+{\beta}_1^k. Treatment+{\beta}_2^k.{BCVA\ level}_t+{\beta}_3^k. phase+{\beta}_4^k. Treatment\ast phase+{\beta}_5^k. phase\ast {BCVA\ level}_t}} $$

In the phakic with cataract population, the probability of being at BCVA level j at month (t + 3) was expressed as a function of treatment, type of lens (phakic with cataract, phakic without cataract, cataract surgery within the last 90 days and pseudophakic/cataract surgery > 90 days), center point retinal thickness (< 400 μm or ≥ 400 μm), BCVA severity level in previous visit, phase, interaction between treatment and cataract, interaction between treatment and phase, and interaction between BCVA severity level in previous visit and phase.

$$ Prob\left({BCVA\ level}_{t+3}=j\ |\ x\right)=\frac{e^{\beta_0^j+{\beta}_1^j. Treatment+{\beta}_2^k. Lens+{\beta}_3^k. CPT+{\beta}_4^j.{BCVA\ level}_t+{\beta}_5^j. phase+{\beta}_6^j. reatment\ast phase+{\beta}_7^j. phase\ast {BCVA\ level}_t}}{1+\sum \limits_{k=1}^{J-1}{e}^{\beta_0^k+{\beta}_1^k. Treatment+{\beta}_2^k. Lens+{\beta}_3^k. CPT+{\beta}_4^k.{BCVA\ level}_t+{\beta}_5^k. phase+{\beta}_6^k. Treatment\ast phase+{\beta}_7^k. phase\ast {BCVA\ level}_t}} $$

For each subgroup and treatment, two transition matrices were produced using the logistic regression models: one for the period from baseline to three months (efficacy phase) and one for the period from 3 months to 6 years (maintenance phase). The sham arm of the FAME trial was considered representative of real-world effectiveness of usual care, even though the utilisation of anti-VEGFs was less frequent in the FAME trial than in current routine practice. We considered this a reasonable assumption, because our target population consisted of patients insufficiently responsive to anti-VEGFs in the study eye.

#### Transition probabilities for dexamethasone in both eyes and usual care in the fellow eye

A network meta-analysis (NMA) was performed to estimate differences in number of letters gained between treatments.

Consistent with previously published models [15, 16], transition probabilities were obtained using the odds-ratios of improving by ≥10 letters vs. laser (for anti-VEGFs), or vs. the FAc implant (for dexamethasone) calibrated to ensure that the mean change in BCVA predicted by the model at 24 months was equal to the NMA results. Transition probabilities for usual care in the fellow eye were obtained as weighted average of probabilities for dexamethasone, anti-VEGFs and laser photocoagulation (represented by the sham arm of FAME). Details of the NMA are provided in the Additional file 2.

All-cause mortality was age- and gender-dependent and was taken from the 2012–2014 lifetable for the UK published by the Office for National Statistics (ONS) [18].

### Costs and health outcomes estimates

Costs were estimated from the perspective of the National Health Service (NHS) and Personal Social Services (PSS). Costs considered in the model included drug, treatment administration, disease monitoring, adverse event management and blindness costs. Treatment costs were extracted from the British National Formulary (BNF) [19] and the NHS Dictionary of Medicines and Devices (DM + D) [20]. Monitoring costs and the costs of treating adverse events were sourced from NHS Reference Costs (2015–16) [21]. Costs of blindness were estimated based on published literature and applied to patients with a BCVA < 35 letters in both eyes [22].

The effect of the FAc implant and alternative treatments was estimated in terms of quality-adjusted life-years (QALYs), which consider both extension of lifespan and quality of life. Health state utilities linked to BCVA levels in both eyes were based on existing models [16, 23]. The original source was a time-trade off study in AMD [24]. Utility decrements for adverse events were obtained from the literature [25]. The impact of long-term adverse effects of treatment that affects BCVA (e.g. cataract, glaucoma) was assumed to be captured in BCVA levels modelled based on clinical trial data.

Treatment with anti-VEGFs has been reported to cause anxiety directly connected with treatment injections [26]. Taking this into account, the disutility connected with anxiety was included in the model and applied during treatment with anti-VEGFs.

Model inputs (demographics, transition probabilities, costs and utilities) are presented in Table 2. Costs and outcomes were discounted at 3.50% per year in line with NICE recommendations [27].

**Table 2:** Key model inputs

	Base case	Low value	High value	Source
Patients characteristics				
Starting age – phakic	63.80	56.24	71.36	ICE-UK
Starting age – pseudophakic	68.50	66.90	70.10	ICE-UK
Proportion of males	61.4%	54.0%	68.5%	ICE-UK
Percentage of patients with DMO affecting both eyes at baseline	76.6%	70.0%	82.6%	ICE-UK
Percentage of FE with a cataract at baseline	27.2%	18.1%	37.3%	ICE-UK
Percentage of pseudophakic FE at baseline	52.6%	45.1%	60.1%	ICE-UK
Baseline distribution of BCVA levels	ICE-UK			
Probabilities^b^				
Probability of developing DMO in the fellow eye, per cycle^a^	5.4%	2.4%	9.7%	ICE-UK
Probability of developing a cataract in the FE	6.8%	4.7%	9.2%	FAME data, assumed to be the same as for laser photocoagulation [17]
Probability of retreatment with FAc implant	35.2%	24.5%	48.9%	Based on FAME data on ≥15 letter improvement in BCVA [17]
Probability of retreatment with dexamethasone	66.7%	48.2%	82.8%	Mastropasqua et al., 2015 [37]
Probability of receiving study treatment in the FE	30.4%	22.2%	39.1%	ICE-UK
Probability of cataract surgery – change of intercept used within the logit model	Logit model on FAME data depending on the treatment phase and BCVA level			
Mortality				
Relative mortality risk (diabetes)	1.95	1.64	2.33	Preis et al. – 2009 [38]
Relative mortality risk (DMO)	1.23	1.16	1.31	Christ et al. – 2008 [39], Ranibizumab Technology Appraisal Guidance 2013 Section 13.4, page 13. [40]
Transition probabilities				
Transition Matrices between different BCVA levels for FAc implant and laser photocoagulation^c^	Multinomial logistic model applied to patient-level data from the ICE-UK study for pseudophakic patients and the full study population.			
Odds -ratio of BCVA improvement for dexamethasone vs. FAc implant^b^	0.90	0.60	1.43	Calibration method, based on the NMA results for mean change in BCVA letter score from baseline to 24 months for pseudophakic population with chronic DMO
Odds-ratio of BCVA improvement for anti-VEGFs vs. laser photocoagulation^b^	2.36	2.15	2.60	
Probability of worsening to lower BCVA level during the end-of-life phase^b^	3.5%	2.3%	5.0%	NICE ERG report for aflibercept [25]
Usual care treatment mix				
Laser photocoagulation	28.3%	20.3%	38.2%	ICE-UK, low and high value defined for laser, for the rest of treatments calculated proportionally to the base case scenario
Ranibizumab 0.5 mg/injection	62.6%	69.6%	53.9%	
Bevacizumab 1.25 mg/injection	9.1%	10.1%	7.8%	
Aflibercept 2 mg/injection	0%	0%	0%	
Number of drug administrations per treatment regimen				
FAc 0.2 μg/day implant [per 3 years]	1	–	–	FAc 0.2 μg/day implant SPC [9]
Laser photocoagulation [per year]	0.98	0.74	1.23	NICE ERG report of aflibercept [25]
Usual Care [per year]	1.68	1.31	2.05	ICE-UK
Dexamethasone [per 6 months]	1	–	–	Dexamethasone SPC, conservative assumption [8]
Drug costs				
FAc 0.2 μg/day implant	£5500.00	–	–	Alimera Science
Ranibizumab	£551.00	£330.60	–	DM + D
Bevacizumab	£242.66	£145.60	–	BNF (January 2017)
Aflibercept	£816.00	£489.60	–	BNF (January 2017)
Dexamethasone	£870.00	£522.00	–	BNF (January 2017)
Health state utilities				
Utilities depended on the BCVA levels	According to the study by Czoski-Murray et al. 2009 [24], worse BCVA level was related to lower health state utility. Both eyes were considered when determining utility depending on visual acuity: values varied from 0.8559 for both eyes with the highest possible visual acuity, to 0.2634 for both blind eyes. Baseline utility in the model was estimated to be 0.63 and 0.66 in the pseudophakic and phakic population, respectively.			
Utility decrements				
Retinal detachment repair	0.130	0.079	0.181	Evidence Review Group report for aflibercept submission (p. 82) [25]
Vitreous haemorrhage	0.020	0.012	0.028	
Disutility for anxiety associated with injections applied during treatment with anti-VEGFs	0.071	0.043	0.099	UK population norms EQ-5D [41]
Percentage of patients with anxiety while on treatment with anti-VEGFs	0.173	0.133	0.218	Senra et.al. 2017 [26]

^a^For patients with DMO affecting the study eye only

^b^Probabilities fixed over time in the model

^c^Probabilities differentiated between efficacy and maintenance phase, i.e. 0 to 3 months or after 3 months

*Anti-VEGF* Anti-Vascular Endothelial Growth Factor, *BCVA* Best-Corrected Visual Acuity, *BNF* British National Formulary, *DM + D* Dictionary of Medicines and Devices, *DMO* Diabetic Macular Oedema, *ERG* Evidence Review Group, *FAc* Fluocinolone Acetonide, *FE* Fellow Eye, *ICE-UK* ILUVIEN Clinical Evaluation-UK, *NICE* National Institute for Health and Care Excellence, *NMA* Network Meta-Analysis, *SPC* Summary of Product Characteristics

### Analyses

Costs incurred over the 15-year time horizon were reported per patient for each treatment strategy. Although QALYs were the final health outcome measure used in the model, the improvement in visual acuity (expressed as the average number of ETDRS letters gained [28]) at 3 and 36 months was also calculated. Predicted number of anti-VEGF injections avoided over 6 years after FAc implant administration was also reported.

The incremental cost per QALY gained for FAc implant vs. comparators (ICER) was calculated as the final result of the model. Deterministic sensitivity analyses (DSA) were conducted by replacing base-case values with upper and lower limits of the 95% confidence intervals. Probabilistic sensitivity analysis (PSA) was based on 1000 iterations.

## Results

### FAc 0.2 μg/day implant compared with usual care in patients who were pseudophakic at baseline

**Fig. 3:**
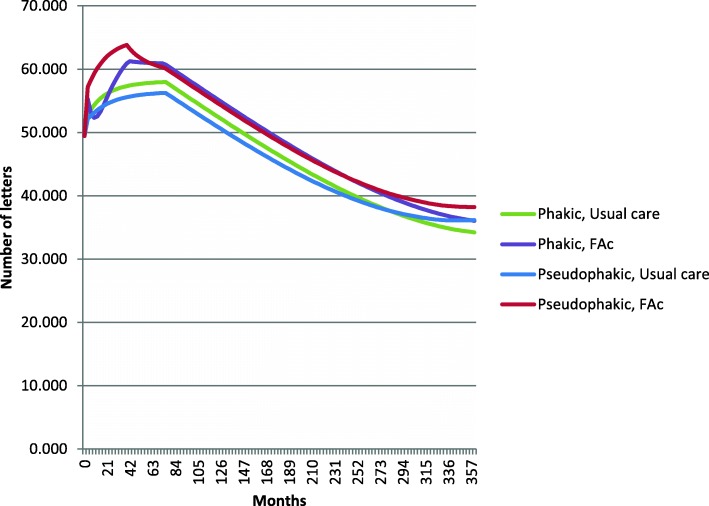
Average predicted BCVA scores for FAc 0.2 μg/day implant and usual care in the pseudophakic and phakic populations

The average number of ETDRS letters gained following treatment with the FAc 0.2 μg/day implant in the study eye was 7.73 at 3 months and 10.87 at 36 months. The differences in visual gain in the study eye between the FAc implant and UC were 5.00 and 4.01 letters at 3 and 36 months, respectively (Fig. 3). The estimated number of anti-VEGF injections avoided was 6.27 over six years of treatment with the FAc implant.

The average number of QALYs in patients treated with the FAc 0.2 μg/day implant was 5.776 over the 15-year model time horizon – an additional 0.185 QALYs compared with UC, which was associated with 5.592 QALYs. Total costs per patient over 15 years were £3066 higher for the FAc 0.2 μg/day implant than for UC. Thus, the incremental cost-effectiveness ratio (ICER) vs. UC for the FAc 0.2 μg/day implant was £16,609 per QALY gained. We also calculated the maximum price at which the FAc implant would be cost-effective considering a £30,000 willingness-to-pay (WTP) threshold; this price (hereafter referred to as threshold price) was £7183.

In line with the assumption that treatment duration could not exceed 6 years (Table 1), all drug, monitoring and administration costs were incurred within the first six years in the model (Fig. 5), during which patients received treatment. The costs of adverse events and blindness were incurred throughout the 15-year time horizon of the model. For the study eye, total drug costs (including all administered treatments) were higher for the FAc implant than for UC (£7982 vs. £3237 per patient). For the fellow eye, both drugs (£5580 vs. £5193) and adverse event management (£1935 vs. £1432) per-patient costs were slightly higher for the FAc implant compared with UC. However, monitoring costs, administration costs and the costs associated with blindness were lower with the FAc implant than with usual care. The estimates of different cost types for all treatment strategies are provided in Table 3.

**Table 3:** Predicted costs and QALYs by treatment strategy and population, per patient over 15 years

	Pseudophakic population		Phakic population		
	FAc 0.2 μg/day implant	Usual Care	Dexamethasone	FAc 0.2 μg/day implant	Usual care
Costs					
Drug – SE, of which:	£7982	£3237	£4841	£8078	£3330
Corticosteroids^a^	£7092	£0	£2478	£7140	£0
Other treatments	£890	£3237	£2363	£938	£3330
Drug – FE	£5580	£5193	£5319	£5822	£5348
Corticosteroids^a^	£986	£0	£364	£1235	£0
Other treatments	£4594	£5193	£4955	£4587	£5348
Monitoring – SE	£4538	£6366	£5944	£4676	£6550
Monitoring – FE	£525	£551	£544	£535	£567
Adverse event	£1935	£1432	£1415	£3666	£3095
Blindness	£114	£194	£163	£188	£227
Administration	£1442	£2078	£2114	£1460	£2139
Total costs	£22,117	£19,051	£20,340	£24,425	£21,255
QALYs	5.78	5.59	5.65	6.36	6.25

^a^FAc or dexamethasone

**Fig. 4:**
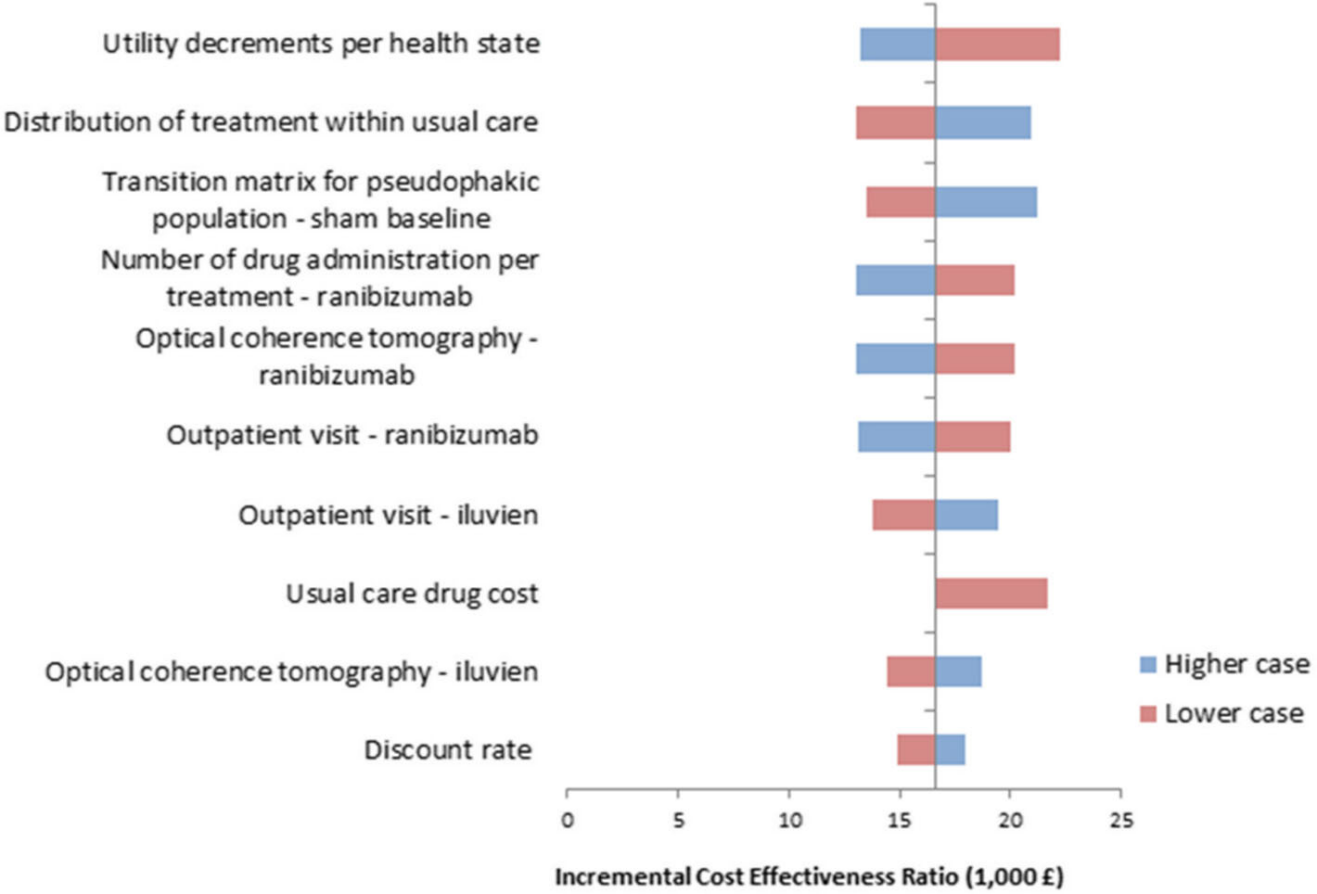
Results of deterministic sensitivity analyses for FAc 0.2 μg/day implant vs. usual care in the pseudophakic population

**Fig. 5:**
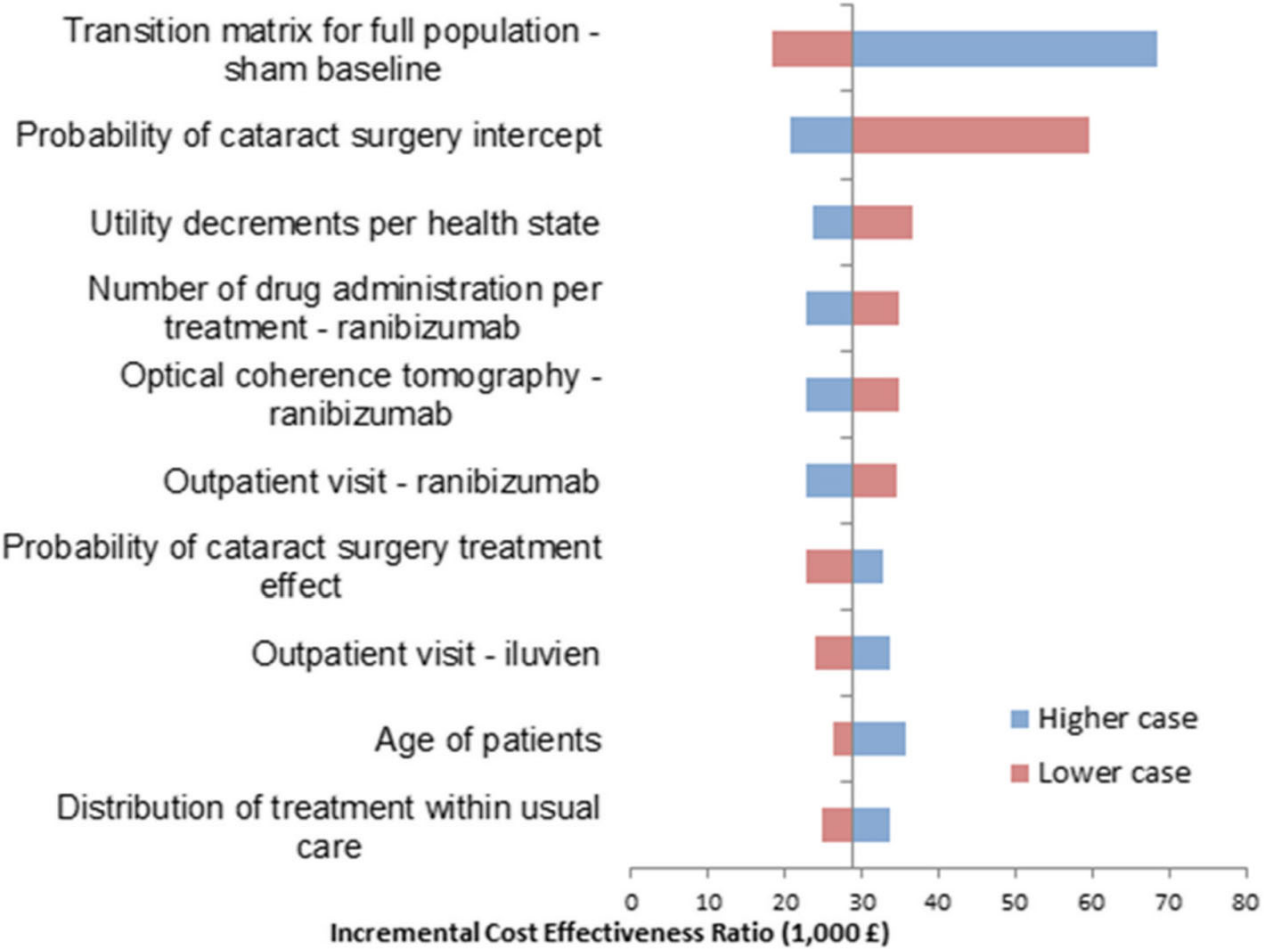
Results of deterministic sensitivity analyses for FAc 0.2 μg/day implant vs. usual care in the phakic population

**Fig. 6:**
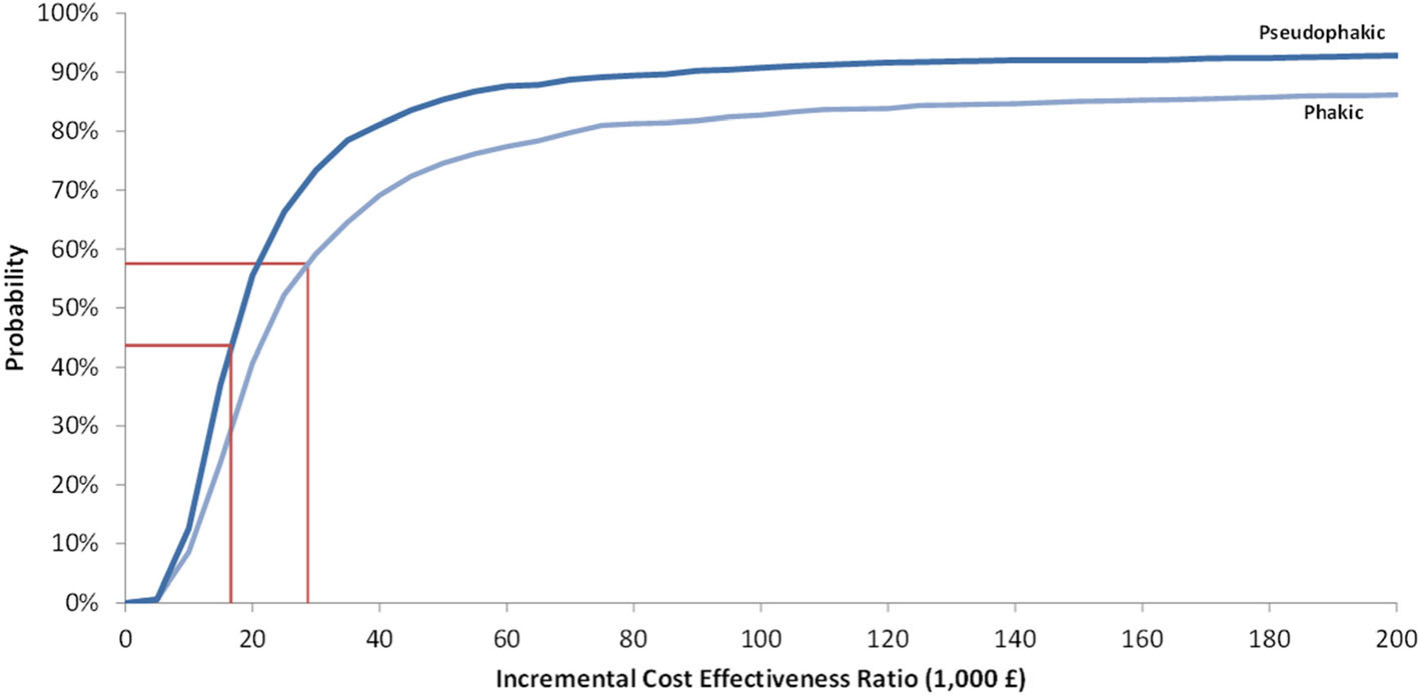
Cost-effectiveness acceptability curves for FAc 0.2 μg/day implant vs. usual care in pseudophakic and phakic populations

DSA showed that the main incremental cost-effectiveness ratio (ICER) drivers included utility decrements per health state, distribution of treatment within usual care, transition probabilities and parameters connected with the use of ranibizumab (Figs. 4 and 5). The results of the PSA obtained from 1000 simulations are illustrated by the cost-effectiveness plane (Additional file 3: Figure S3 and Additional file 4: Figure S4) and the cost-effectiveness acceptability curve (Fig. 6). The probability of the FAc implant being cost-effective vs. UC was 73.4% at a willingness-to-pay threshold of £30,000 per QALY gained.

### FAc 0.2 μg/day implant compared with dexamethasone in patients who were pseudophakic at baseline

The modelled visual gain with the dexamethasone implant was 6.46 letters at 3 months and 7.24 letters at 36 months – less than the gain with the FAc implant in the same pseudophakic population (see above). Indeed, the differences in visual gains in the study eye between the FAc implant and dexamethasone were 0.28 and 3.64 ETDRS letters in favour of the FAc implant at 3 and 36 months, respectively **(**Additional file 5: Figure S2**)**.

The FAc 0.2 μg/day implant strategy was predicted to associate with 0.126 additional QALYs over 15 years compared with the dexamethasone implant (5.776 vs. 5.650 QALYs, respectively) at an additional cost of £1777 (£22,117 vs. £20,340, respectively). Thus, the incremental cost per QALY gained (or ICER) for the FAc 0.2 μg/day implant vs. dexamethasone implant was £14,070 applying the full NHS price. We also computed the threshold price of the FAc implant, which amounted to £6870 at a WTP of £30,000 per QALY. DSA showed that the main ICER drivers were costs of dexamethasone and of outpatient visits for FAc-treated patients (Additional file 6: Figure S5).

As mentioned above, total costs per patient over 15 years were higher by £1777 for the FAc implant compared with dexamethasone. When different cost types were considered (Table 3), total drug costs for the study eye were higher for FAc implant than for dexamethasone implant (£7982 vs. £4841). Drug cost for the fellow eye and costs of adverse event management were also slightly higher for the FAc implant. On the other hand, disease monitoring costs, administration costs and costs associated with blindness were lower for the FAc implant than for dexamethasone implant.

### FAc 0.2 μg/day implant vs. usual care in the population of patients who were phakic at baseline

Phakic patients treated with the FAc 0.2 μg/day implant gained an average of 5.87 ETDRS letters at 3 months and 11.46 at 36 months. The differences in visual gains in the study eye between FAc 0.2 μg/day implant and UC were 2.59 and 2.96 letters at 3 and 36 months respectively. The predicted number of anti-VEGF injections avoided over 6 years after FAc 0.2 μg/day implant administration was 6.69.

The number of QALYs associated with the FAc 0.2 μg/day implant over 15 years was 6.36, providing 0.11 additional QALYs compared with UC, which was associated with 6.25 QALYs over the same time frame. The additional cost associated with the FAc implant compared with UC was £3170 over the 15-year time horizon (total cost of £24,425 vs. £21,255), resulting in an ICER of £28,751 per QALY gained. In this setting, the threshold price of the FAc implant was £5590 at a WTP threshold of £30,000 per QALY.

Total drug costs for the study eye were substantially higher for the FAc 0.2 μg/day implant than for usual care (£8078 vs. £3330). Similarly to the results obtained in pseudophakic patients, drug costs for the fellow eye and the costs of adverse event management were slightly higher with the FAc implant than with UC. However, monitoring, administration and blindness costs were lower with the FAc 0.2 μg/day implant than with usual care. Different cost types for both treatment strategies are presented in Table 3.

DSA showed that the main ICER drivers were transition probabilities (Figs. 4 and 5). PSA results (Fig. 6) showed that the probability of the FAc 0.2 μg/day implant being cost-effective was 59.2% at a willingness-to-pay threshold of £30,000 per QALY gained.

### Scenario analysis

In addition to the sensitivity analysis, two additional scenarios were analysed: scenario A, in which the baseline distributions by BCVA level were switched between study eye and fellow eye so that the study eye was more likely to be the best-seeing eye, and Scenario B, in which UC included laser photocoagulation only and BCVA was assumed to be constant during that treatment. Scenario A resulted in ICERs equal to £17,022 and £36,023 in the pseudophakic and phakic population, respectively, compared to usual care. At a WTP of £30,000/QALY, threshold prices of the FAc 0.2 μg/day implant amounted to £7094 in the pseudophakic population and £5150 in the phakic population **(**Additional file 7: Table S1). Scenario B, where no anti-VEGFs were used within UC and BCVA remained constant, resulted in an ICER for the FAc 0.2 μg/day implant vs. usual care of £29,622 and £30,792 in the pseudophakic and phakic population, respectively. In this scenario, threshold prices of the FAc implant were £5581 and £5338, respectively in the pseudophakic and phakic populations **(**Additional file 8: Table S2). All parameters of sensitivity analyses are presented in Additional file 9: Table S3.

## Discussion

Our analysis investigated cost-effectiveness of the FAc 0.2 μg/day implant in patients who suffered from chronic DMO in at least one eye that was pseudophakic or phakic with a cataract, and did not respond sufficiently, or ceased to respond sufficiently, to anti-VEGF treatments. With a base-case ICER of £16,609 and £28,751 per QALY in pseudophakic and phakic patients, the FAc 0.2 μg/day implant was estimated to be cost-effective compared with usual care, considering that a cost-effectiveness threshold of £20,000–£30,000 per QALY is normally applied in the UK [27]. Thus, the FAc 0.2 μg/day implant can provide a cost-effective solution also for phakic patients, whose treatment options are currently quite limited. The results of sensitivity analyses were generally consistent with the base-case scenario. However, the ICER estimate was sensitive to the assumption that anti-VEGFs continue to be used in patients insufficiently responsive to these therapies, as seen in Scenario B, where patients were assumed to receive laser photocoagulation only and their vision neither improved nor deteriorated with this treatment. Nonetheless, the inclusion of anti-VEGFs in usual care of patients that do not respond to them sufficiently appears justified, as available clinical trial evidence suggest patients may continue treatment with anti-VEGFs despite insufficient response to the initial phase of treatment [13, 29, 30]. Further, with the dosing regimen of anti-VEGFs based on the real-world ICE-UK study, the assumptions used to model anti-VEGF treatment are more likely to accurately reflect the reality of using these treatments on the NHS.

The FAc 0.2 μg/day implant was estimated to be cost-effective compared with dexamethasone in patients who were pseudophakic at baseline, with a base-case ICER of £14,070. Furthermore, with a price reduction of £1210, the FAc 0.2 μg/day implant would dominate the dexamethasone implant. It is worth noting that this comparison against dexamethasone is likely to be highly conservative, as we assumed retreatment with the dexamethasone implant could be performed every six months in line with the approved indication [8], while its dosing in routine clinical practice is likely to be more frequent (i.e. every 3–4 months) [31], thus increasing the costs associated with this treatment option. This assumption was consistent with the efficacy data we used, which was derived from a clinical study of the dexamethasone implant [32], where treatment frequency was in line with the label.

Our model appeared to provide a reasonable approximation of the changes in visual acuity observed in the FAME trial, which provides reassurance on the validity of the modelling approach, inputs and assumptions. In the model, pseudophakic patients treated with the FAc 0.2 μg/day implant gained 10.9 ETDRS letters over 3 years, while phakic patients gained 11.5 letters – an overestimation of 3.3 and 3.9 letters, respectively, compared with the FAME trial, where the average number of ETDRS letters gained with the FAc 0.2 μg/day implant was 7.6 [17]. It is, however, worth noting that this improvement in visual acuity in the FAME trial was measured in a population with a mixed lens status – 54.5% of chronic DMO patients treated with the FAc implant and 58.9% of those treated with sham injection were phakic at baseline [17]. In the usual care group, the model predicted a gain of 6.8 ETDRS letters in pseudophakic patients and 8.5 ETDRS letters in phakic patients, compared with a gain of 1.8 letters reported in the sham arm of the FAME trial [17] – an overestimate of 6.7 letters. Although the model seems to overestimate gains in BCVA obtained with both treatments, it can be considered conservative, as the degree of overestimation was higher in the usual care arm, and the modelled benefit of the FAc implant (4.0 and 3.0 additional ETDRS letters gained with the FAc implant compared with usual care at 3 years in pseudophakic and phakic patients, respectively) was lower than that observed in the FAME trial (between-treatment difference of 6.1 letters at 3 years [17]). The difference between BCVA observed in the FAME trial and BCVA predicted in the model appears attributable to random variability – although probabilities of transition between BCVA categories estimated using a more complex multinomial model allowed for a better fit to the trial data, some factors were removed from the model since they were not statistically significant.

In terms of structure, the current model is similar to other UK models for DMO treatments – two such models were identified, one used in the dexamethasone implant Single Technology Appraisal (STA), the other in STA of aflibercept. In the model used within aflibercept STA, patients treated with laser photocoagulation accumulated 7.30 QALYs (11.34 life-years [LY]) over a lifetime horizon [14]. In order to compare our results with those presented in aflibercept STA submission, we performed an additional analysis extending the time horizon of the present model to 30 years. The analysis showed that patients treated with laser photocoagulation lived on average 9.66 years, corresponding to 6.05 QALYs. This difference between QALY estimates between the two models results from differences in mortality that was included. However, the ratio between generated QALYs and LYs is consistent between both models.

It is noteworthy that results of the comparison between dexamethasone implant and the FAc 0.2 μg/day implant were largely similar in the current model and the model used in the dexamethasone implant STA. Over a 15-year time horizon, the dexamethasone STA model estimated that total costs were £22,365 for the FAc implant compared with £20,413 for the dexamethasone implant [33]. In our model, the same comparison performed in the pseudophakic population resulted in total costs of £22,117and £20,340 for the two treatments, respectively. The results of both models were also comparable in terms of QALY estimates, with the model used in dexamethasone implant STA returning 5.82 QALYs with the FAc implant compared to 5.74 QALYs with the dexamethasone implant [33], and the current model estimating 5.78 and 5.65 QALYs, respectively.

### Study limitations

Generalisability of our results may be somewhat affected by the fact that it is uncertain whether the outcomes of the sham arm in the FAME trial are indeed representative for current usual care provided to patients insufficiently responsive to anti-VEGFs across the UK. Although the 1.8-letter mean change from baseline at 36 months seen in patients with chronic DMO treated with the sham control [17] seems to represent poor response, it may be more than could be expected in an insufficiently responsive population. Indeed, for some patients who failed to respond to usual care, the visual acuity could progressively decline over 3 years. For that reason, using the outcomes of the sham arm from FAME to describe treatment without sufficient efficacy can be considered conservative.

Another limitation of the study relates to uncertainty around some of the inputs, in particular the utilities, which were based on a study in patients with AMD, not DMO. However, sensitivity analyses suggested that the results were robust. Another limitation related to input data was the fact we were unable to use Patient Access Scheme prices within the model, as this type of information is commercially confidential. Finally, an NMA was used to estimate the relative effectiveness of the dexamethasone and FAc 0.2 μg/day implants; the main limitation of this analysis was the use of studies differing in patient characteristics, which generally included wider populations than that modelled in the present study.

Further limitations arise from some assumptions made to simplify the model, such as independence of the two eyes assumed when calculating utilities and cost of blindness, a simplified schedule of injections (e.g. with the FAc 0.2 μg/day implant injections possible only at baseline or 3 years in both the study and the fellow eye), and the fact all patients stopped treatment after 6 years and BCVA was assumed to decline at a constant rate after that. In addition, the duration of the model cycle was 3 months, which meant that using treatment intervals ranging between 3 and 6 months would complicate the structure of the model. This particularly affected the dexamethasone implant, for which a dosing frequency in that range may be used in clinical practice [31].

Finally, using the FAc 0.2 μg/day implant, which is an effective therapy administered as a single injection up to every 36 months, may help to reduce burden on the hospitals, with fewer intravitreal injections and monitoring visits compared with other available treatments [34]. The reduction in injections and visits may not only benefit the healthcare system, but also avoid patient compliance issues, as DMO patients experience a heavy treatment burden, compounded by anxiety related to intravitreal injections [34]. Although the current model did capture the number of intravitreal injections avoided and accounted for injection-related anxiety, we did not investigate the effects of treatment on patient compliance or indirect costs related to DMO treatment, such as those of travel to clinic appointments.

Overall, the results of this model support the use of the FAc 0.2 μg/day implant as a cost-effective treatment option in patients with chronic DMO that is inadequately responsive to available therapies. This includes phakic patients in whom the benefits of the FAc implant appear evident, although the availability of a PAS should further ensure the price falls within the UK WTP threshold of £20,000–30,000 per QALY. Indeed, the results of this model are broadly in line with an earlier report from a UK NHS hospital, where the use of the FAc 0.2 μg/day implant was shown to produce cost savings [12].

## Conclusion

The FAc 0.2 μg/day implant was estimated to be cost-effective compared to usual care in patients with chronic DMO who display an insufficient response to anti-VEGFs irrespective of the lens status. Further, the FAc 0.2 μg/day implant was cost-effective compared to dexamethasone implant in patients with pseudophakic eyes, for whom both of these treatments are currently recommended.

## Additional files

Additional file 1: **Figure S1.** Treatment strategies in the model – study and fellow eye. (DOCX 36 kb)

Additional file 2: NMA SLR supplement Supplementary materials explaining the methods used in the systematic literature review, the network meta-analysis, and the statistical study to compare the clinical efficacy of Illuvien® and pharmacological treatments for DME by performing a Bayesian mixed treatment comparison of randomized controlled trials. (DOCX 220 kb)

Additional file 3: **Figure S3.** Incremental cost-effectiveness plane – FAc 0.2 μg/day implant vs. usual care in pseudophakic population. (DOCX 44 kb)

Additional file 4: **Figure S4.** Incremental cost-effectiveness plane – FAc 0.2 μg/day implant vs. usual care in phakic population. (DOCX 98 kb)

Additional file 5: **Figure S2.** Average BCVA score for FAc 0.2 μg/day implant and dexamethasone (study and fellow eyes, pseudophakic population). (DOCX 38 kb)

Additional file 6: **Figure S5.** Results of deterministic sensitivity analyses, FAc 0.2 μg/day implant vs. dexamethasone in pseudophakic population. (DOCX 31 kb)

Additional file 7: **Table S1.** Results of Scenario A. (DOCX 13 kb)

Additional file 8: **Table S2.** Results of Scenario B. (DOCX 13 kb)

Additional file 9: **Table S3.** Parameters for sensitivity analyses. (DOCX 58 kb)

## Abbreviations

anti-VEGFs: Intravitreal vascular endothelial growth factor inhibitors
BCVA: Best-corrected visual acuity
BNF: British National Formulary
DM + D: Dictionary of Medicines and Devices
DMO: Diabetic macular oedema
DRCR: Diabetic Retinopathy Clinical Research
DSA: Deterministic sensitivity analyses
ERG: Evidence Review Group
ETDRS: Early Treatment Diabetic Retinopathy Study
FAc: Fluocinolone acetonide
FE: Fellow Eye
ICERs: Incremental cost-effectiveness ratios
ICE-UK: ILUVIEN Clinical Evaluation-UK
LY: Life-years
NHS: National Health Service
NICE: National Institute for Health and Care Excellence
NMA: Network meta-analysis
ONS: Office for National Statistics
PSA: Probabilistic sensitivity analysis
PSS: Personal Social Services
QALYs: Quality-adjusted life years
SPC: Summary of Products Characteristics
STA: Single Technology Appraisal
UC: Usual care
WTP: Willingness-to-pay
