# Supplementary material for: Cost-effectiveness of fluocinolone acetonide implant (ILUVIEN®) in UK patients with chronic diabetic macular oedema considered insufficiently responsive to available therapies
Source: BMC Health Serv Res. 2019 Jan 9;19:22. doi: 10.1186/s12913-018-3804-4 (PMC6327492; doi:10.1186/s12913-018-3804-4)
Supplement: Supplementary file 1 — Figure S1. Treatment strategies in the model – study and fellow eye. (DOCX 36 kb) [file 12913_2018_3804_MOESM1_ESM.docx]

Additional file 1: Figure S1.
