# Supplementary material for: Cost-effectiveness of fluocinolone acetonide implant (ILUVIEN®) in UK patients with chronic diabetic macular oedema considered insufficiently responsive to available therapies
Source: BMC Health Serv Res. 2019 Jan 9;19:22. doi: 10.1186/s12913-018-3804-4 (PMC6327492; doi:10.1186/s12913-018-3804-4)
Supplement: Supplementary file 2 — NMA SLR supplement Supplementary materials explaining the methods used in the systematic literature review, the network meta-analysis, and the statistical study to compare the clinical efficacy of Illuvien® and pharmacological treatments for DME by performing a Bayesian mixed treatment comparison of randomized controlled trials. (DOCX 220 kb) [file 12913_2018_3804_MOESM2_ESM.docx]

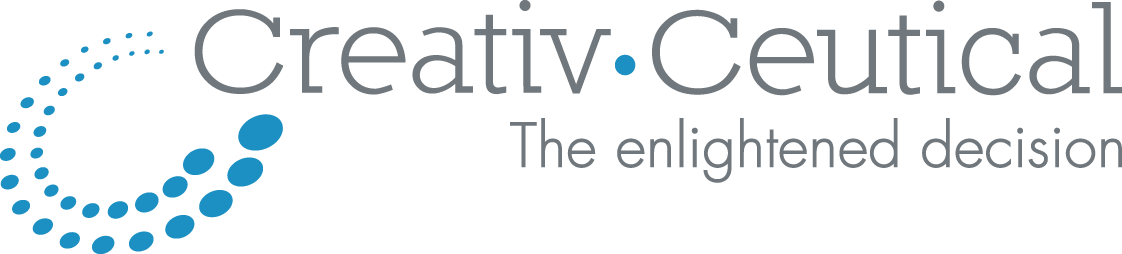


|  | **Cost-effectiveness of fluocinolone acetonide implant (ILUVIEN®) in UK patients with diabetic macular oedema insufficiently responsive to other therapies**  **Supplementary materials - systematic literature review and network meta-analysis** |
| --- | --- |
|  |  |
|  |  |
|  |  |
|  |  |
|  |  |
|  |  |
|  |  |

# Objective

To compare the clinical efficacy of fluocinolone acetonide intravitreal implant (Illuvien®) and pharmacological treatments for diabetic macular edema (DME) by performing a Bayesian mixed treatment comparison of randomized controlled trials.

# Methods

## Systematic literature review

- The systematic literature review was undertaken according to the Centre for Review sand Dissemination and the Preferred Reporting Items for Systematic Review and Meta-analysis

**Literature search and studies selection**

- The literature searches were performed on May 2016 using the following bibliographical databases and service providers: Ovid MEDLINE(R) In-Process & Other Non-Indexed Citations and Ovid MEDLINE(R), EMBASE (Ovid), and Cochrane CENTRAL (Central Register of Controlled) Trials database
- The review was performed targeting RCTs with the following criteria: studies on adult patients (18 years old and more) treated for DME, evaluating the efficacy, safety or quality of life outcome measures of DME pharmacological treatments.
- The list of unique titles and abstracts was screened by two independent reviewers to select relevant articles according to the defined inclusion and exclusion criteria. The two lists of selected references were then compared and differences were resolved by consensus. Non-relevant papers were excluded with the reasons for exclusion documented.

**Data extraction and quality control**

- Studies meeting the criteria for inclusion were presented in the form of Excel-based extraction tables. Two reviewers independently extracted the data from the included studies. A quality control of the completed extraction grids was performed by a third investigator.
- Extracted data included the study characteristics, information on treatment arms and population, efficacy outcomes (BCVA, retinal thickness) and safety outcomes.
- When necessary, data were extracted from figures using Grafula software.

**Quality assessment**

- The quality assessment of the included RCTs was conducted according to the CRD's guidance for undertaking reviews in health care (University of York Centre for Reviews and Dissemination). [1]
- The studies included in the review were assessed for randomization, treatment allocation, blinding and quality of the analyses.
- The quality assessment was performed by two reviewers in dependently. Disagreements were resolved by consensus.

## Network meta-analysis

- In order to assess the relative efficacy of the interventions of interest, Bayesian network meta-analyses were performed. Fixed and random effects models were used.
- Prior to mixed treatment comparisons, additional eligibility criteria outlined in was applied to the selected studies in order to confirm a final set of studies of relevance to the decision problem that could be used to populate the mixed treatment comparison network.
- For efficacy outcomes, data were analysed using intention-to-treat (ITT) results obtained with last observation carried forward (LOCF) or Markov chain Monte Carlo method of multiple imputation methods from the included studies. For safety outcomes, per protocol population data was used.
- The mean change in BCVA letters on the ETDRS scale at 24 months was assessed.
- Results for the chronic and pseudophakic patients at 24 months were included in the economic evaluation. As data for the chronic subgroup were available for the FAc 0.2 µg implant, no restriction based on duration of disease was used for the other treatments. In addition, since no data for the pseudophakic subgroup were available for anti-VEGFs, no restriction based on lens status was used for anti-VEGFs.

Table 1: Inclusion and exclusion criteria for the SLR studies selection and the MTC analysis

| PICOS elements | Inclusion | Exclusion |
| --- | --- | --- |
| Population | - Adults (aged more than 18 years old) with DME   **Additional inlusion criteria for the MTC:**   - Intention to treat (ITT), Full analysis set (FAS), modified- ITT ((m-)ITT), safety (per protocol) population | - Children (<18 years) - Macular oedema not associated with diabetes uveitis |
| Intervention | - Fluocinolone acetonide (ILUVIEN®) - laser photocoagulation - dexamethasone (OZURDEX®) - Anti-VEGFs: aflibercept (EYLEA®) or bevacizumab (AVASTIN®) or ranibizumab (LUCENTIS®)) - Combination of the above treatment | - Acupuncture, herbal therapy, dietary, or alternative medicine |
| Comparator | - Fluocinolone acetonide (ILUVIEN®) - laser photocoagulation - dexamethasone (OZURDEX®) - Anti-VEGFs: aflibercept (EYLEA®) or bevacizumab (AVASTIN®) or ranibizumab (LUCENTIS®)) - Combinations of the above treatments only, placebo or sham treatment | - Non relevant comparators |
| Outcomes* | - Efficacy: - BCVA (mean change from baseline, mean average change from baseline, as measured by ETDRS score or Snellen equivalent) - Loss of ≥ 10, ≥ 15, ≥ 30 ETDRS letters - Gain of ≥ 0, 10, 15, 30 ETDRS letters - 20/40 vision or better (Snellen chart) - 20/200 or worse (Snellen chart) - Reduction in laser use - Anatomical changes (e.g., change in CNV and lesion area, central foveal thickness, and fluid on OCT) - Health-related quality of life (EQ-5D, NEI VFQ-25, and other scales) - Safety: treatment withdrawals, moderate to severe adverse events | - Non relevant outcomes |
| Study designs | - Randomized controlled trials (RCTs) - SLR of RCTs - Meta-analysis of RCTs - Pooled study of RCTs   **Additional inclusion criteria for the MTC:**   - LOCF imputation or Markov chain Monte Carlo method of multiple imputation - Estimates of mean changes in BVCA with SD, SE, CI, or p values - Estimates of probabilities of events | - Study not controlled or not randomized - Retrospective study - Pharmacokinetic study - Economic or cost study - Review (systematic or not) of economic models or economic studies - Editorial, Letter to Editor, Opinion, clinical review or trial protocol - Study on quality of life outcomes only - Not systematic review of clinical trials - Case study - Study duration ≤ 6 months   **Additional exclusion criteria for the MTC:**   - Observed data - No available estimates |
| *Several outcomes were considered in the SLR. However, we only report results related to the mean change in BCVA at24 months in this document. | | |

## Statistical methods

- Bayesian NMA models were used to simultaneously synthesize the results of the included studies for each outcome of interest.[2-5] Both fixed effects (FE) and random effects (RE) models [3, 5, 6] were assessed, and were compared using the deviance information criterion (DIC).
- A normal likelihood distribution was assumed for the mean change in BCVA.
- Results are presented in a graphical format, using a forest plot.
- The analyses were performed using WinBUGS 1.4.1 statistical software. The WinBUGS programs were based on the NICE decision Support Unit TSD document. [7]

# Results

## Studies selection

- In total 2727 references where identified: 965 hits were identified from Medline database, 1242 from Embase, and 520 from Cochrane. After duplicates removing, 1792 records were screened (title and abstract), of them, 176 references were retained for full text screening to end up with 51 relevant publications. Finally, a total of 21 publications were eligible for the network meta-analysis. The cumulative number of randomized eyes was 7145. 8 trials met the eligibility criteria for the mean change in BCVA letter score at 24 months analysis in chronic pseudophakic subgroup.

Figure 1: PRISMA diagram indicating the selection process for the network meta-analysis

**Records identified through database searching**

**(n=2727)**

Medline (n=965)

Embase (n=1242)

Cochrane (n=520)

**Duplicates removed
(n=927)**

)

)

**Identification**

**Records excluded
(n=1624)**

)

)

**Records screened
(n=1800)**

)

**Screening**

**Full-text articles excluded**

**(n=130)**

Not relevant population (n=9); Not relevant intervention (n=21); Not relevant comparator (n=1); Not relevant outcome (n=12); Study not controlled or not randomized (n=14); Editorial, Letter to Editor, Opinion, clinical review or trial protocol (n=11); Study on quality of life outcomes only (n=1); Not systematic review of clinical trials (n=31); Study duration ≤ 6 months (n=7); Duplicate (n=9); SLR/NMA/MTC (n=14)

**Full-text articles assessed for eligibility
(n=176)**

**Eligibility**

**Records identified from other sources**

**(n=5)**

Abstracts (n=4); Trial reports provided by the manufacturer (n = 1)

**Studies included in the qualitative analysis**

**(n=51)**

**Included**

**Studies included in the quantitative analysis (NMA)**

**(n =21)**

## Studies characteristics

- Overall, 18 trials (21 publications) were eligible for NMA selection criteria
- A risk-of-bias assessment was conducted using the CRD's guidance for undertaking reviews in health care). Most information was from studies at low risk of bias.

Table 2: Overview of the selected studies in the NMA

| Trial name | Country | Design | Population | Intervention | Comparator | Trial length | Nb of randomized eyes | Publications |
| --- | --- | --- | --- | --- | --- | --- | --- | --- |
| BEVORDEX (NCT01298076) | Australia and UK | Phase II, prospective, multicentre, randomized, single-masked clinical trial | DMO affecting the central fovea | Bevacizumab 1.25 mg | Dexamethasone 0.7 mg | 12 months | 61 | Gillies et al. 2014 [8] |
| BOLT (eudract.ema.europa.eu Identifier: 2007-000847-89) | UK | Prospective, randomized, masked, single-centre, 2-year, 2-arm clinical trial. | Persistent clinically significant macular oedema and at least one previous laser treatment | Bevacizaumab 1.25 mg | Laser | 24 months | 80 | Rajendram et al. 2012 [9], Michaelides et al. 2010 [10] |
| DA VINCI (NCT00789477) | USA, Canada, and Austria | Randomized, double-masked, active-controlled multicentre phase 2 clinical trial | Centre-involved DMO | Aflibercept 0.5q4  Aflibercept 2q4  Aflibercept 2q8  Aflibercept 2PRN | Laser | 12 months | 221 | Do et al. 2012 [11] |
| FAME A and B (NCT00344968) | FAME A: United States, Canada, European Union, and India  FAME B: United States, India, European Union | Two parallel, prospective, randomized, sham injection-controlled, double-masked, multicenter, 36-month studies clinical trials | Persistent DMO despite ≥1 macular laser treatment | Fluocinolone acetonide 0.2-mcg/day  Fluocinolone acetonide 0.5-mcg/day | Sham | 36 months | 956 | Cunha-Vaz et al. 2014 [12], Campochiaro 2012 [13], Campochiaro 2011 [14]  Report from the manufacturer [15] |
| MEAD (NCT00168337 and NCT00168389) | Worldwide | Two randomized, multicentre, masked, sham-controlled, phase III clinical trials with identical protocols | DMO | Dexamethasone 0.35 mg  Dexamethasone 0.7 mg | Sham | 3 years | 1048 | Boyer et al. 2014 [16] |
| Protocol I (NCT00445003) | USA | Phase III, randomized, multicentre clinical trial | DMO involving the fovea | Ranibizumab 0.5 mg + Deferred Laser  Ranibizumab 0.5 mg + Prompt Laser | Sham + Prompt Laser | 3 years | 854 | Elman et al. 2011 [17] |
| NCT00490815 | USA | Phase II, prospective, randomized, interventional, multicenter clinical trial | Persistent DMO despite > or = 1 focal/grid laser therapy | Fluocinolone acetonide 0.2-mcg/day | Fluocinolone acetonide 0.5-mcg/day | 12 months | 37 | Campochiaro et al. 2010 [18] |
| Protocol T (NCT01627249) | USA, Canada | Multicentre, randomized, phase III clinical trial | DMO involving the macular centre | Aflibercept 2.0 mg | Bevacizumab 1.25 mg  Ranibizumab 0.3 mg | 2 years | 660 | Wells et al. 2015 [19] |
| OZLASE (EudraCT 2011-003339-74) | UK | Phase III, single-centre, prospective, randomised, active-controlled trial | Centre-involving DMO | Dexamethasone + Laser | Laser | 56 weeks | 80 | Heng et al. 2015 [20] |
| PLACID (NCT00464685) | USA, Canada | Randomized, controlled, multicentre, double-masked, parallel-group, 12-month, phase III trial | Diffuse DMO | Dexamethasone 0.7 mg + Laser | Laser | 1 year, with up to 3 months of additional follow-up | 253 | Callanan et al. 2013 [21] |
| RESOLVE (NCT00284050) | Switzerland | 12-month, randomized, controlled, double-masked, multicentre, phase II study | DMO involving the foveal centre | Ranizumab 0.3mg-0.6mg  Ranizumab 0.5mg-1.0mg | Sham | 12 months | 151 | Massin et al. 2010 [22] |
| RESTORE (NCT00687804) | 10 European countries, Turkey, Canada, and Australia | 12-month, phase III, randomized, double-masked, multicentre, laser-controlled study | DMO | Ranibizumab 0.5 mg  Ranibizumab 0.5 mg + Laser | Laser | 12 months | 345 | Mitchell et al. 2011 [23] |
| RETAIN (NCT01171976) | International | 24-month, phase IIIb, single-masked, controlled, three-arm parallel-group study | DMO | T&E ranibizumab 0.5 mg+laser  T&E ranibizumab 0.5 mg | PRN ranibizumab 0.5 mg | 2 years | 372 | Prunte et al. 2016 [24] |
| REVEAL (NCT00989989) | China, Hong Kong, Japan, South Korea, Singapore, Taiwan | 12-month, randomized, double-masked, laser-controlled, multicentre, phase III study | DMO (Asian patients) | Ranibizumab 0.5 mg  Ranibizumab 0.5 mg + laser | Laser | 12 months | 396 | Ishibashi et al. 2015 [25] |
| RIDE and RISE (NCT00473382, NCT00473330) | United States and South America | Methodologically identical, phase III, randomized, multicentre, double-masked, trials that were sham injection controlled for the first 2 years | Centre-involving DMO | Ranibizumab 0.3 mg  Ranibizumab 0.5 mg | Sham | 36 months | 759 | Nguyen et al. 2012 [26] |
| VISTA and VIVID (NCT01363440, NCT01331681) | VISTA: USA  VIVID: Europe, Japan, Australia | Two similarly designed double-masked, randomized, active-controlled, 148-week, phase III trials | Centre-involving DMO | Aflibercept 2q4  Aflibercept 2q8 | Laser | 148 weeks | VISTA: 466  VIVID: 406 | Brown et al. 2015 [27], Korobelnik et al. 2014 [28], Do et al. 2016 [29] |

## Mean change from baseline in BCVA letter score

Figure 2: Forest plots for the mean change in BCVA letter score predicted from baseline to 24 months for chronic pseudophakic patients. The error bar indicates the 95% credibility intervals of the estimate.

| **Chronic pseudophakic patients population – fixed effects model** ****  AF= Aflibercept; BEV= Bevacisumab; RAN= ranibizumab; DEX= Dexamethasone ; FA= Fluocinolone acetonide |
| --- |
|  |

# Discussion

- The efficacy of fluocinolone acetonide against sham in the treatment of DMO has been demonstrated in the clinical trials. However, no data on the relative efficacy of fluocinolone acetonide compared to other available treatments are available. Consequently, a NMA of randomized controlled trials was performed in order to compare the clinical efficacy and safety of fluocinolone acetonide intravitreal implant and pharmacological treatments for DMO in different subgroups of interest.
- First limitation of this study is that the number of available clinical trials remains limited. Ranibizumab is the most studied drug with a higher number of available clinical trials, followed by aflibercept. Only one RCT which studied fluocinolone acetonide was considered in the analysis. There was an uncertainty regarding the quality of the included studies and, consequently, potential bias. This mainly concerns randomisation methods and allocation concealment which were poorly described by authors. In addition, 10 of 29 identified studies were not double-masked, and for another six trials the blinding is unclear.
- The analysis is also limited by the heterogeneity of the included studies. Patients’ baseline characteristics were generally not well reported in the publications, making it difficult to compare patient populations between the studies. Study design might also differ across the trials, especially, in terms of treatment regimen. For instance, a number of studies authorised rescue laser therapy together with studied drugs, while others did not. Significant differences are to be noted between FAME studies where any type of additional therapy could be used (including anti-VEGFs) and other trials where no additional therapy or only rescue laser was available for patients with poor response. Studies that required patient to exit the trial if other therapy was administrated (such as MEAD studies) were associated with a high discontinuation rate. Although the design of FAME studies was closer to real-life practice, it could have led to underestimation of the therapeutic effect of fluocinolone acetonide.
- A number of the study assumptions related to the subgroup analyses, that had to be made in the absence of relevant data on subgroups, may also limit the results. First, equal efficacy was considered in chronic and non-chronic DMO for all treatments but fluocinolone acetonide, due to the lack of reported data for chronic subgroups. However, as mentioned previously some studies such RISE and RIDE trials [26, 30-32], as well as RESTORE trial [23] suggested lower efficacy of anti-VEGF treatments in chronic patients. In addition, although fluocinolone acetonide 0.2 mcg is used in patients previously treated with anti-VEGFs with insufficient response in real world, there are no RCT data on the efficacy of anti-VEGFs in such patients, and the reported analyses for patients previously treated with anti-VEGFs assumed equal efficacy between previously treated and anti-VEGF naïve patients for fluocinolone acetonide and dexamethasone. Moreover, an equal efficacy in phakic and pseudophakic patients was assumed for anti-VEGFs relative to laser. However, lens status and cataract formation may have a significant impact on visual acuity independently of DMO progression. Since lens status was poorly reported in the selected studies and might differ considerably between the studies, the overall results may have also been biased.

# References

1. Reviews, C.f.R.a.D.S., *CRD’s guidance for undertaking reviews in health care. .* 2009.

2. Caldwell, D.M., A.E. Ades, and J.P. Higgins, *Simultaneous comparison of multiple treatments: combining direct and indirect evidence.* BMJ, 2005. **331**(7521): p. 897-900.

3. Jansen, J.P., et al., *Bayesian meta-analysis of multiple treatment comparisons: an introduction to mixed treatment comparisons.* Value Health, 2008. **11**(5): p. 956-64.

4. Jansen, J.P., et al., *Interpreting indirect treatment comparisons and network meta-analysis for health-care decision making: report of the ISPOR Task Force on Indirect Treatment Comparisons Good Research Practices: part 1.* Value Health, 2011. **14**(4): p. 417-28.

5. Lu, G. and A.E. Ades, *Combination of direct and indirect evidence in mixed treatment comparisons.* Stat Med, 2004. **23**(20): p. 3105-24.

6. Dias, S., et al., *Evidence synthesis for decision making 2: a generalized linear modeling framework for pairwise and network meta-analysis of randomized controlled trials.* Med Decis Making, 2013. **33**(5): p. 607-17.

7. Dias, S., et al., in *A Generalised Linear Modelling Framework for Pairwise and Network Meta-Analysis of Randomised Controlled Trials*. 2014: London.

8. Gillies, M.C., et al., *A randomized clinical trial of intravitreal bevacizumab versus intravitreal dexamethasone for diabetic macular edema: the BEVORDEX study.* Ophthalmology, 2014. **121**(12): p. 2473-81.

9. Rajendram, R., et al., *A 2-year prospective randomized controlled trial of intravitreal bevacizumab or laser therapy (BOLT) in the management of diabetic macular edema: 24-month data: report 3.* Arch Ophthalmol, 2012. **130**(8): p. 972-9.

10. Michaelides, M., et al., *A prospective randomized trial of intravitreal bevacizumab or laser therapy in the management of diabetic macular edema (BOLT study) 12-month data: report 2.* Ophthalmology, 2010. **117**(6): p. 1078-1086 e2.

11. Do, D.V., et al., *One-year outcomes of the da Vinci Study of VEGF Trap-Eye in eyes with diabetic macular edema.* Ophthalmology, 2012. **119**(8): p. 1658-65.

12. Cunha-Vaz, J., et al., *Sustained delivery fluocinolone acetonide vitreous implants: long-term benefit in patients with chronic diabetic macular edema.* Ophthalmology, 2014. **121**(10): p. 1892-903.

13. Campochiaro, P.A., et al., *Sustained delivery fluocinolone acetonide vitreous inserts provide benefit for at least 3 years in patients with diabetic macular edema.* Ophthalmology, 2012. **119**(10): p. 2125-32.

14. Campochiaro, P.A., et al., *Long-term benefit of sustained-delivery fluocinolone acetonide vitreous inserts for diabetic macular edema.* Ophthalmology, 2011. **118**(4): p. 626-635 e2.

15. Sciences, A., *Additional analyses from FAME trial*. 2016.

16. Boyer, D.S., et al., *Three-year, randomized, sham-controlled trial of dexamethasone intravitreal implant in patients with diabetic macular edema.* Ophthalmology, 2014. **121**(10): p. 1904-14.

17. Elman, M.J., et al., *Expanded 2-year follow-up of ranibizumab plus prompt or deferred laser or triamcinolone plus prompt laser for diabetic macular edema.* Ophthalmology, 2011. **118**(4): p. 609-14.

18. Campochiaro, P.A., et al., *Sustained ocular delivery of fluocinolone acetonide by an intravitreal insert.* Ophthalmology, 2010. **117**(7): p. 1393-9 e3.

19. Diabetic Retinopathy Clinical Research, N., et al., *Aflibercept, bevacizumab, or ranibizumab for diabetic macular edema.* N Engl J Med, 2015. **372**(13): p. 1193-203.

20. Heng, L.Z., et al., *A prospective randomised controlled clinical trial comparing a combination of repeated intravitreal Ozurdex and macular laser therapy versus macular laser only in centre-involving diabetic macular oedema (OZLASE study).* Br J Ophthalmol, 2016. **100**(6): p. 802-7.

21. Callanan, D.G., et al., *Dexamethasone intravitreal implant in combination with laser photocoagulation for the treatment of diffuse diabetic macular edema.* Ophthalmology, 2013. **120**(9): p. 1843-51.

22. Massin, P., et al., *Safety and efficacy of ranibizumab in diabetic macular edema (RESOLVE Study): a 12-month, randomized, controlled, double-masked, multicenter phase II study.* Diabetes Care, 2010. **33**(11): p. 2399-405.

23. Mitchell, P., et al., *The RESTORE study: ranibizumab monotherapy or combined with laser versus laser monotherapy for diabetic macular edema.* Ophthalmology, 2011. **118**(4): p. 615-25.

24. Prunte, C., et al., *Ranibizumab 0.5 mg treat-and-extend regimen for diabetic macular oedema: the RETAIN study.* Br J Ophthalmol, 2016. **100**(6): p. 787-95.

25. Ishibashi, T., et al., *The REVEAL Study: Ranibizumab Monotherapy or Combined with Laser versus Laser Monotherapy in Asian Patients with Diabetic Macular Edema.* Ophthalmology, 2015. **122**(7): p. 1402-15.

26. Nguyen, Q.D., et al., *Ranibizumab for diabetic macular edema: results from 2 phase III randomized trials: RISE and RIDE.* Ophthalmology, 2012. **119**(4): p. 789-801.

27. Brown, D.M., et al., *Intravitreal Aflibercept for Diabetic Macular Edema: 100-Week Results From the VISTA and VIVID Studies.* Ophthalmology, 2015. **122**(10): p. 2044-52.

28. Korobelnik, J.F., et al., *Intravitreal aflibercept for diabetic macular edema.* Ophthalmology, 2014. **121**(11): p. 2247-54.

29. Do, D.V., et al., *Intravitreal Aflibercept Injection in Diabetic Macular Edema Patients with and without Prior Anti-Vascular Endothelial Growth Factor Treatment: Outcomes from the Phase 3 Program.* Ophthalmology, 2016. **123**(4): p. 850-7.

30. Ip, M.S., et al., *Long-term effects of therapy with ranibizumab on diabetic retinopathy severity and baseline risk factors for worsening retinopathy.* Ophthalmology, 2015. **122**(2): p. 367-74.

31. Bressler, N.M., et al., *Vision-related function after ranibizumab treatment for diabetic macular edema: results from RIDE and RISE.* Ophthalmology, 2014. **121**(12): p. 2461-72.

32. Brown, D.M., et al., *Long-term outcomes of ranibizumab therapy for diabetic macular edema: the 36-month results from two phase III trials: RISE and RIDE.* Ophthalmology, 2013. **120**(10): p. 2013-22.
