# Supplementary figures and images for: Cost-effectiveness of fluocinolone acetonide implant (ILUVIEN®) in UK patients with chronic diabetic macular oedema considered insufficiently responsive to available therapies
Source: BMC Health Serv Res. 2019 Jan 9;19:22. doi: 10.1186/s12913-018-3804-4 (PMC6327492; doi:10.1186/s12913-018-3804-4)

Additional file 3: Figure S3

*


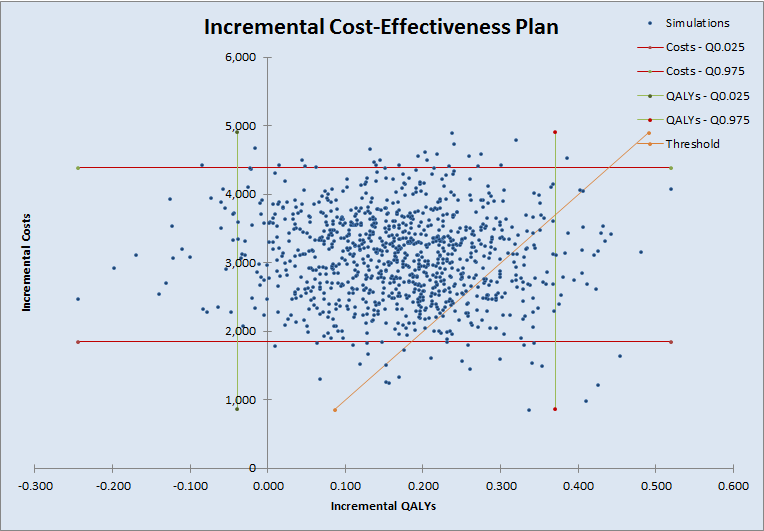

Supplement: Supplementary file 3 — Figure S3. Incremental cost-effectiveness plane – FAc 0.2 μg/day implant vs. usual care in pseudophakic population. (DOCX 44 kb) [file 12913_2018_3804_MOESM3_ESM.docx]

Additional file 5: Figure S2


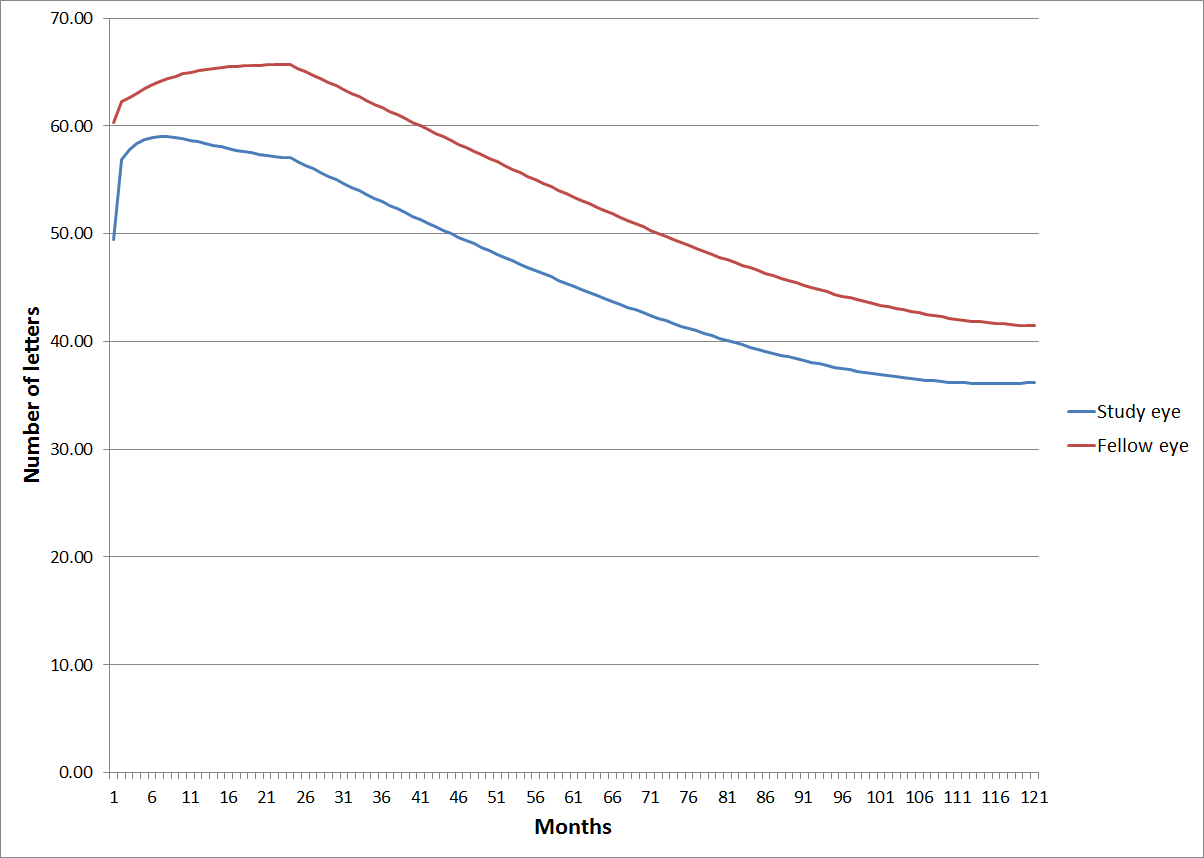

Supplement: Supplementary file 5 — Figure S2. Average BCVA score for FAc 0.2 μg/day implant and dexamethasone (study and fellow eyes, pseudophakic population). (DOCX 38 kb) [file 12913_2018_3804_MOESM5_ESM.docx]

**
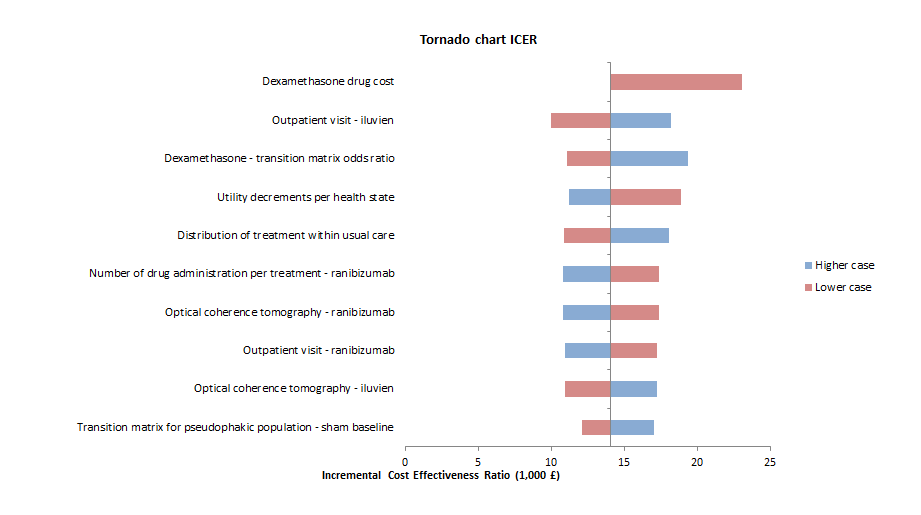
**Additional file 6: **Figure S5.**

*

Supplement: Supplementary file 6 — Figure S5. Results of deterministic sensitivity analyses, FAc 0.2 μg/day implant vs. dexamethasone in pseudophakic population. (DOCX 31 kb) [file 12913_2018_3804_MOESM6_ESM.docx]
