# Supplementary material for: Cost-effectiveness of fluocinolone acetonide implant (ILUVIEN®) in UK patients with chronic diabetic macular oedema considered insufficiently responsive to available therapies
Source: BMC Health Serv Res. 2019 Jan 9;19:22. doi: 10.1186/s12913-018-3804-4 (PMC6327492; doi:10.1186/s12913-018-3804-4)
Supplement: Supplementary file 4 — Figure S4. Incremental cost-effectiveness plane – FAc 0.2 μg/day implant vs. usual care in phakic population. (DOCX 98 kb) [file 12913_2018_3804_MOESM4_ESM.docx]

Additional file 4: Figure S4
