# Supplementary material for: Cost-effectiveness of fluocinolone acetonide implant (ILUVIEN®) in UK patients with chronic diabetic macular oedema considered insufficiently responsive to available therapies
Source: BMC Health Serv Res. 2019 Jan 9;19:22. doi: 10.1186/s12913-018-3804-4 (PMC6327492; doi:10.1186/s12913-018-3804-4)
Supplement: Supplementary file 7 — Table S1. Results of Scenario A. (DOCX 13 kb) [file 12913_2018_3804_MOESM7_ESM.docx]

Additional file 7: Table S1

|  | Pseudophakic population | | | Phakic population | | |
| --- | --- | --- | --- | --- | --- | --- |
|  | FAc 0.2 µg/day implant | Usual Care | Dexamethasone | | FAc 0.2 µg/day implant | Usual care |
| Costs |  |  |  | |  |  |
| Drug – SE | £7,982 | £3,238 | £4,841 | | £8,079 | £3,331 |
| Drug – FE | £5,580 | £5,193 | £5,319 | | £5,822 | £5,347 |
| Monitoring – SE | £4,539 | £6,368 | £5,945 | | £4,677 | £6,551 |
| Monitoring – FE | £525 | £551 | £544 | | £535 | £567 |
| Adverse event | £1,937 | £1,435 | £1,417 | | £3,658 | £3,078 |
| Blindness | £113 | £185 | £159 | | £188 | £216 |
| Administration | £1,442 | £2,078 | £2,114 | | £1,460 | £2,139 |
| Total costs | **£22,118** | **£19,048** | **£20,340** | | **£24,418** | **£21,229** |
| QALYs | **5.7912** | **5.6108** | **5.6670** | | **6.3561** | **6.2676** |
