# Supplementary material for: Cost-effectiveness of fluocinolone acetonide implant (ILUVIEN®) in UK patients with chronic diabetic macular oedema considered insufficiently responsive to available therapies
Source: BMC Health Serv Res. 2019 Jan 9;19:22. doi: 10.1186/s12913-018-3804-4 (PMC6327492; doi:10.1186/s12913-018-3804-4)
Supplement: Supplementary file 8 — Table S2. Results of Scenario B. (DOCX 13 kb) [file 12913_2018_3804_MOESM8_ESM.docx]

Additional file 8: Table S2

|  | Pseudophakic population | | Phakic population | |
| --- | --- | --- | --- | --- |
|  | FAc 0.2 µg/day implant | Usual Care | FAc 0.2 µg/day implant | Usual care |
| Costs |  |  |  |  |
| Drug – SE | £7,982 | £0 | £8,078 | £0 |
| Drug – FE | £5,580 | £5,193 | £5,822 | £5,348 |
| Monitoring – SE | £4,538 | £3,625 | £4,676 | £3,729 |
| Monitoring – FE | £525 | £551 | £535 | £567 |
| Adverse event | £1,935 | £1,431 | £3,666 | £3,062 |
| Blindness | £114 | £299 | £188 | £364 |
| Administration | £1,442 | £1,705 | £1,460 | £1,755 |
| Total costs | **£22,117** | **£12,804** | **£24,425** | **£14,825** |
| QALYs | **5.7762** | **5.4618** | **6.3599** | **6.0482** |
