# Supplementary material for: Cost-effectiveness of fluocinolone acetonide implant (ILUVIEN®) in UK patients with chronic diabetic macular oedema considered insufficiently responsive to available therapies
Source: BMC Health Serv Res. 2019 Jan 9;19:22. doi: 10.1186/s12913-018-3804-4 (PMC6327492; doi:10.1186/s12913-018-3804-4)
Supplement: Supplementary file 9 — Table S3. Parameters for sensitivity analyses. (DOCX 58 kb) [file 12913_2018_3804_MOESM9_ESM.docx]

Additional file 9: Table S3

|  | Base case | DSA | | | | PSA | | | Source | |  |
| --- | --- | --- | --- | --- | --- | --- | --- | --- | --- | --- | --- |
|  |  | Low value | High value | | | Distribution | Parameters | |  |  |  |
| Discount rate | | | | | | | | | | |  |
| Discount rate – Costs | 3.5% | 1.5% | 5.0% | | | - | - | | NICE, assumption | |  |
| Discount rate – QALY | 3.5% | 1.5% | 5.0% | | | - | - | |  |  |  |
| Baseline characteristics | | | | | | | | | | |  |
| Starting age – phakic | 63.80 | 56.24 | 71.36 | | | Normal (µ,σ) | µ=63.800, σ=3.859 | | ICE-UK, 95% CI | |  |
| Starting age – pseudophakic | 68.50 | 66.90 | 70.10 | | | Normal (µ,σ) | µ =68.50, σ=0.814 | |  |  |  |
| Proportion of male | 61.4% | 54.0% | 68.5% | | | Beta (µ,σ) | µ=0.614, σ=0.0374 | |  |  |  |
| Population (fellow eye) at baseline | | | | | | | | | | |  |
| % of patients with bilateral disease | 76.6% | 70.0% | 82.6% | | | Beta (µ,σ) | µ=0.766, σ=0.032 | | ICE-UK, 95% CI | |  |
| % of FE with cataract | 27.2% | 18.1% | 37.3% | | | Beta (µ,σ) | µ=0.272, σ=0.049 | |  |  |  |
| % of pseudophakic FE | 52.6% | 45.1% | 60.1% | | | Beta (µ,σ) | µ=0.526, σ=0.039 | |  |  |  |
| Study Eye BCVA Distribution | | | | | | | | | | |  |
| With DMO | | | | | | | | | | |  |
| Baseline distribution for pseudophakic and phakic with cataract population | | | | | | | | | | |  |
| 86 – 100 | 0.6% | 0.0% | 4.1% | | | Dirichlet_n  (α1, …,αn) | α1=1  α2=4  α3=29  α4=40  α5=30  α6=26  α7=9  α8=21 | | BC: ICE UK; DSA: FAME (worse BCVA) and ICE-UK fellow eye (better BCVA); PSA: Dirichlet distribution based on ICE-UK study eye | |  |
| 76 – 85 | 2.5% | 0.0% | 17.8% | | |  |  |  |  |  |  |
| 66 – 75 | 18.1% | 12.8% | 26.0% | | |  |  |  |  |  |  |
| 56 – 65 | 25.0% | 36.9% | 16.4% | | |  |  |  |  |  |  |
| 46 – 55 | 18.8% | 22.7% | 6.8% | | |  |  |  |  |  |  |
| 36 – 45 | 16.3% | 14.2% | 12.3% | | |  |  |  |  |  |  |
| 26 – 35 | 5.6% | 9.2% | 1.4% | | |  |  |  |  |  |  |
| 25 – 0 | 13.1% | 4.3% | 15.1% | | |  |  |  |  |  |  |
| Fellow Eye BCVA Distribution | | | | | | | | | | |  |
| Without DMO | | | | | | | | | | |  |
| Baseline distribution for phakic without cataract population | | | | | | | | | | |  |
| 86 – 100 | 8.7% | 0.0% | 39.1% | | | Dirichlet_n  (α1, …,αn) | α1=2  α2=7  α3=4  α4=3  α5=6  α6=0  α7=0  α8=1 | | BC: ICE-UK; DSA: all categories shifted down (worse BCVA) or up (better BCVA) by 1 level; PSA: Dirichlet distribution based on ICE-UK study eye | |  |
| 76 – 85 | 30.4% | 8.7% | 17.4% | | |  |  |  |  |  |  |
| 66 – 75 | 17.4% | 30.4% | 13.0% | | |  |  |  |  |  |  |
| 56 – 65 | 13.0% | 17.4% | 26.1% | | |  |  |  |  |  |  |
| 46 – 55 | 26.1% | 13.0% | 0.0% | | |  |  |  |  |  |  |
| 36 – 45 | 0.0% | 26.1% | 0.0% | | |  |  |  |  |  |  |
| 26 – 35 | 0.0% | 0.0% | 4.3% | | |  |  |  |  |  |  |
| 25 – 0 | 4.3% | 4.3% | 0.0% | | |  |  |  |  |  |  |
| Baseline distribution for phakic with cataract population | | | | | | | | | | |  |
| 86 – 100 | 17.6% | 0.0% | 47.1% | | | Dirichlet_n  (α1, …,αn) | α1=3  α2=5  α3=1  α4=4  α5=4  α6=0  α7=0  α8=0 | | BC: ICE-UK; DSA: all categories shifted down (worse BCVA) or up (better BCVA) by 1 level; PSA: Dirichlet distribution based on ICE-UK study eye | |  |
| 76 – 85 | 29.4% | 17.6% | 5.9% | | |  |  |  |  |  |  |
| 66 – 75 | 5.9% | 29.4% | 23.5% | | |  |  |  |  |  |  |
| 56 – 65 | 23.5% | 5.9% | 23.5% | | |  |  |  |  |  |  |
| 46 – 55 | 23.5% | 23.5% | 0.0% | | |  |  |  |  |  |  |
| 36 – 45 | 0.0% | 23.5% | 0.0% | | |  |  |  |  |  |  |
| 26 – 35 | 0.0% | 0.0% | 0.0% | | |  |  |  |  |  |  |
| 25 – 0 | 0.0% | 0.0% | 0.0% | | |  |  |  |  |  |  |
| Baseline distribution for pseudophakic population | | | | | | | | | | |  |
| 86 – 100 | 17.6% | 0.0% | 47.1% | | | Dirichlet_n  (α1, …,αn) | α1=3  α2=5  α3=1  α4=4  α5=4  α6=0  α7=0  α8=0 | | Assumed the same as phakic | |  |
| 76 – 85 | 29.4% | 17.6% | 5.9% | | |  |  |  |  |  |  |
| 66 – 75 | 5.9% | 29.4% | 23.5% | | |  |  |  |  |  |  |
| 56 – 65 | 23.5% | 5.9% | 23.5% | | |  |  |  |  |  |  |
| 46 – 55 | 23.5% | 23.5% | 0.0% | | |  |  |  |  |  |  |
| 36 – 45 | 0.0% | 23.5% | 0.0% | | |  |  |  |  |  |  |
| 26 – 35 | 0.0% | 0.0% | 0.0% | | |  |  |  |  |  |  |
| 25 – 0 | 0.0% | 0.0% | 0.0% | | |  |  |  |  |  |  |
| With DMO | | | | | | | | | | |  |
| Baseline distribution for phakic without cataract population | | | | | | | | | | |  |
| 86 – 100 | 10.3% | 0.0% | 31.0% | | | Dirichlet_n  (α1, …,αn) | α1=6  α2=12  α3=13  α4=6  α5=8  α6=3  α7=5  α8=5 | | BC: ICE-UK; DSA: ICE-UK - study eye (worse BCVA) all categories shifted down up by 1 level (better BCVA); PSA: Dirichlet distribution based on ICE-UK study eye | |  |
| 76 – 85 | 20.7% | 0.0% | 22.4% | | |  |  |  |  |  |  |
| 66 – 75 | 22.4% | 9.1% | 10.3% | | |  |  |  |  |  |  |
| 56 – 65 | 10.3% | 54.5% | 13.8% | | |  |  |  |  |  |  |
| 46 – 55 | 13.8% | 27.3% | 5.2% | | |  |  |  |  |  |  |
| 36 – 45 | 5.2% | 0.0% | 8.6% | | |  |  |  |  |  |  |
| 26 – 35 | 8.6% | 9.1% | 8.6% | | |  |  |  |  |  |  |
| 25 – 0 | 8.6% | 0.0% | 0.0% | | |  |  |  |  |  |  |
| Baseline distribution for phakic with cataract population | | | | | | | | | | |  |
| 86 – 100 | 4.1% | 0.6% | 21.9% | | | Dirichlet_n  (α1, …,αn) | α1=3  α2=13  α3=19  α4=12  α5=5  α6=9  α7=1  α8=11 | | BC: ICE-UK; DSA: ICE-UK - study eye (worse BCVA) all categories shifted down up by 1 level (better BCVA); PSA: Dirichlet distribution based on ICE-UK study eye | |  |
| 76 – 85 | 17.8% | 2.5% | 26.0% | | |  |  |  |  |  |  |
| 66 – 75 | 26.0% | 18.1% | 16.4% | | |  |  |  |  |  |  |
| 56 – 65 | 16.4% | 25.0% | 6.8% | | |  |  |  |  |  |  |
| 46 – 55 | 6.8% | 18.8% | 12.3% | | |  |  |  |  |  |  |
| 36 – 45 | 12.3% | 16.3% | 1.4% | | |  |  |  |  |  |  |
| 26 – 35 | 1.4% | 5.6% | 15.1% | | |  |  |  |  |  |  |
| 25 – 0 | 15.1% | 13.1% | 0.0% | | |  |  |  |  |  |  |
| Baseline distribution for pseudophakic population | | | | | | | | | | |  |
| 86 – 100 | 4.1% | 0.6% | 21.9% | | | Dirichlet_n  (α1, …,αn) | α1=3  α2=13  α3=19  α4=12  α5=5  α6=9  α7=1  α8=11 | | Assumed the same as phakic | |  |
| 76 – 85 | 17.8% | 2.5% | 26.0% | | |  |  |  |  |  |  |
| 66 – 75 | 26.0% | 18.1% | 16.4% | | |  |  |  |  |  |  |
| 56 – 65 | 16.4% | 25.0% | 6.8% | | |  |  |  |  |  |  |
| 46 – 55 | 6.8% | 18.8% | 12.3% | | |  |  |  |  |  |  |
| 36 – 45 | 12.3% | 16.3% | 1.4% | | |  |  |  |  |  |  |
| 26 – 35 | 1.4% | 5.6% | 15.1% | | |  |  |  |  |  |  |
| 25 – 0 | 15.1% | 13.1% | 0.0% | | |  |  |  |  |  |  |
| Distribution of treatment within usual care | | | | | | | | | | |  |
| Laser photocoagulation | 28.3% | 20.3% | 38.2% | | | Dirichlet_n  (α1, …,αn) | α1=28  α2=62  α3=9  α4=0 | | ICE-UK, low and high value defined for laser, for the rest of treatments calculated proportionally to the base case scenario | |  |
| Ranibizumab | 62.6% | 69.6% | 53.9% | | |  |  |  |  |  |  |
| Bevacizumab | 9.1% | 10.1% | 7.8% | | |  |  |  |  |  |  |
| Aflibercept | 0% | 0% | 0% | | |  |  |  |  |  |  |
| Population – Progression | | | | | | | | | | |  |
| Prob of DMO in FE, per cycle | 5.4% | 2.4% | 9.7% | | | Beta (µ,σ) | µ=0.054, σ=0.019 | | ICE-UK, 20% (SE=0.063) developed DMO within 1 year, of 40 fellow eyes initially not treated for DMO, 95% CI | |  |
| Prob of cataract in FE (no DMO) | 6.8% | 4.7% | 9.2% | | | Beta (µ,σ) | µ=0.068, σ=0.012 | | Assumed to be the same as for laser photocoagulation | |  |
| Prob of cataract in FE (laser) | 6.8% | 4.7% | 9.2% | | | Beta (µ,σ) | µ=0.068, σ=0.012 | | FAME data, 95% CI | |  |
| Prob of cataract in FE (Ran) | 6.8% | 4.7% | 9.2% | | | Beta (µ,σ) | µ=0.068, σ=0.012 | | Assumed to be the same as for laser photocoagulation | |  |
| Prob of cataract in FE (Bev) | 6.8% | 4.7% | 9.2% | | | Beta (µ,σ) | µ=0.068, σ=0.012 | |  |  |  |
| Prob of cataract in FE (Afli) | 6.8% | 4.7% | 9.2% | | | Beta (µ,σ) | µ=0.068, σ=0.012 | |  |  |  |
| Prob of cataract in FE (UC) | 6.8% | 4.7% | 9.2% | | | Beta (µ,σ) | µ=0.068, σ=0.012 | |  |  |  |
| Study Eye - Probability of Retreatment | | | | | | | | | | |  |
| With FAc | | | | | | | | | | |  |
| 86 – 100 | 35.2% | 24.5% | 48.9% | | | Beta (µ,σ) | µ=0.352, σ=0.057 | | BC: Based on probability of 10-letter improvement, estimated from FAME, obtained from post hoc analysis  DSA: min - based on 15 letters improvement from dataset by CPT400; max - 5 letter improvement  PSA: beta distribution based on the BC | |  |
| 76 – 85 | 35.2% | 24.5% | 48.9% | | | Beta (µ,σ) | µ=0.352, σ=0.057 | |  |  |  |
| 66 – 75 | 35.2% | 24.5% | 48.9% | | | Beta (µ,σ) | µ=0.352, σ=0.057 | |  |  |  |
| 56 – 65 | 35.2% | 24.5% | 48.9% | | | Beta (µ,σ) | µ=0.352, σ=0.057 | |  |  |  |
| 46 – 55 | 35.2% | 24.5% | 48.9% | | | Beta (µ,σ) | µ=0.352, σ=0.057 | |  |  |  |
| 36 – 45 | 35.2% | 24.5% | 48.9% | | | Beta (µ,σ) | µ=0.352, σ=0.057 | |  |  |  |
| 26 – 35 | 35.2% | 24.5% | 48.9% | | | Beta (µ,σ) | µ=0.352, σ=0.057 | |  |  |  |
| 25 – 0 | 35.2% | 24.5% | 48.9% | | | Beta (µ,σ) | µ=0.352, σ=0.057 | |  |  |  |
| With dexamethasone | | | | | | | | | | |  |
| 86 – 100 | 66.7% | 48.2% | 82.8% | | | Beta (µ,σ) | µ=0.667, σ=0.089 | | BC: Mastropasqua et al, 2015 (Italy, 27 eyes)  PSA: beta distribution based on BC, 95% CI | |  |
| 76 – 85 | 66.7% | 48.2% | 82.8% | | | Beta (µ,σ) | µ=0.667, σ=0.089 | |  |  |  |
| 66 – 75 | 66.7% | 48.2% | 82.8% | | | Beta (µ,σ) | µ=0.667, σ=0.089 | |  |  |  |
| 56 – 65 | 66.7% | 48.2% | 82.8% | | | Beta (µ,σ) | µ=0.667, σ=0.089 | |  |  |  |
| 46 – 55 | 66.7% | 48.2% | 82.8% | | | Beta (µ,σ) | µ=0.667, σ=0.089 | |  |  |  |
| 36 – 45 | 66.7% | 48.2% | 82.8% | | | Beta (µ,σ) | µ=0.667, σ=0.089 | |  |  |  |
| 26 – 35 | 66.7% | 48.2% | 82.8% | | | Beta (µ,σ) | µ=0.667, σ=0.089 | |  |  |  |
| 25 – 0 | 66.7% | 48.2% | 82.8% | | | Beta (µ,σ) | µ=0.667, σ=0.089 | |  |  |  |
| Fellow Eye | | | | | | | | | | |  |
| Probability of treatment with FAc | | | | | | | | | | |  |
| 86 – 100 | 30.4% | 22.2% | 39.1% | | | Beta (µ,σ) | µ=0.304, σ=0.043 | | BC: ICE-UK (ICE-UK 34 of patients treated with FAc in the FE)  PSA: beta distribution based on BC, 95% CI | |  |
| 76 – 85 | 30.4% | 22.2% | 39.1% | | | Beta (µ,σ) | µ=0.304, σ=0.043 | |  |  |  |
| 66 – 75 | 30.4% | 22.2% | 39.1% | | | Beta (µ,σ) | µ=0.304, σ=0.043 | |  |  |  |
| 56 – 65 | 30.4% | 22.2% | 39.1% | | | Beta (µ,σ) | µ=0.304, σ=0.043 | |  |  |  |
| 46 – 55 | 30.4% | 22.2% | 39.1% | | | Beta (µ,σ) | µ=0.304, σ=0.043 | |  |  |  |
| 36 – 45 | 30.4% | 22.2% | 39.1% | | | Beta (µ,σ) | µ=0.304, σ=0.043 | |  |  |  |
| 26 – 35 | 30.4% | 22.2% | 39.1% | | | Beta (µ,σ) | µ=0.304, σ=0.043 | |  |  |  |
| 25 – 0 | 30.4% | 22.2% | 39.1% | | | Beta (µ,σ) | µ=0.304, σ=0.043 | |  |  |  |
| Probability of retreatment with FAc (after 36 months) | | | | | | | | | | |  |
| 86 – 100 | 35.2% | 24.5% | 48.9% | | | Beta (µ,σ) | µ=0.352, σ=0.057 | | BC: Based on probability of 10-letter improvement, estimated from FAME, obtained from post hoc analysis  DSA: min - based on 15 letters improvement from dataset by CPT400; max - 5 letter improvement  PSA: beta distribution based on the BC | |  |
| 76 – 85 | 35.2% | 24.5% | 48.9% | | | Beta (µ,σ) | µ=0.352, σ=0.057 | |  |  |  |
| 66 – 75 | 35.2% | 24.5% | 48.9% | | | Beta (µ,σ) | µ=0.352, σ=0.057 | |  |  |  |
| 56 – 65 | 35.2% | 24.5% | 48.9% | | | Beta (µ,σ) | µ=0.352, σ=0.057 | |  |  |  |
| 46 – 55 | 35.2% | 24.5% | 48.9% | | | Beta (µ,σ) | µ=0.352, σ=0.057 | |  |  |  |
| 36 – 45 | 35.2% | 24.5% | 48.9% | | | Beta (µ,σ) | µ=0.352, σ=0.057 | |  |  |  |
| 26 – 35 | 35.2% | 24.5% | 48.9% | | | Beta (µ,σ) | µ=0.352, σ=0.057 | |  |  |  |
| 25 – 0 | 35.2% | 24.5% | 48.9% | | | Beta (µ,σ) | µ=0.352, σ=0.057 | |  |  |  |
| Probability of treatment with dexamethasone | | | | | | | | | | |  |
| 86 – 100 | 30.4% | 22.2% | 39.1% | | | Beta (µ,σ) | µ=0.304, σ=0.043 | | Assumed the same as for FAc | |  |
| 76 – 85 | 30.4% | 22.2% | 39.1% | | | Beta (µ,σ) | µ=0.304, σ=0.043 | |  |  |  |
| 66 – 75 | 30.4% | 22.2% | 39.1% | | | Beta (µ,σ) | µ=0.304, σ=0.043 | |  |  |  |
| 56 – 65 | 30.4% | 22.2% | 39.1% | | | Beta (µ,σ) | µ=0.304, σ=0.043 | |  |  |  |
| 46 – 55 | 30.4% | 22.2% | 39.1% | | | Beta (µ,σ) | µ=0.304, σ=0.043 | |  |  |  |
| 36 – 45 | 30.4% | 22.2% | 39.1% | | | Beta (µ,σ) | µ=0.304, σ=0.043 | |  |  |  |
| 26 – 35 | 30.4% | 22.2% | 39.1% | | | Beta (µ,σ) | µ=0.304, σ=0.043 | |  |  |  |
| 25 – 0 | 30.4% | 22.2% | 39.1% | | | Beta (µ,σ) | µ=0.304, σ=0.043 | |  |  |  |
| Probability of retreatment with dexamethasone | | | | | | | | | | |  |
| 86 – 100 | 66.7% | 48.2% | 82.8% | | | Beta (µ,σ) | µ=0.667, σ=0.089 | | Assumed the same as in the study eye | |  |
| 76 – 85 | 66.7% | 48.2% | 82.8% | | | Beta (µ,σ) | µ=0.667, σ=0.089 | |  |  |  |
| 66 – 75 | 66.7% | 48.2% | 82.8% | | | Beta (µ,σ) | µ=0.667, σ=0.089 | |  |  |  |
| 56 – 65 | 66.7% | 48.2% | 82.8% | | | Beta (µ,σ) | µ=0.667, σ=0.089 | |  |  |  |
| 46 – 55 | 66.7% | 48.2% | 82.8% | | | Beta (µ,σ) | µ=0.667, σ=0.089 | |  |  |  |
| 36 – 45 | 66.7% | 48.2% | 82.8% | | | Beta (µ,σ) | µ=0.667, σ=0.089 | |  |  |  |
| 26 – 35 | 66.7% | 48.2% | 82.8% | | | Beta (µ,σ) | µ=0.667, σ=0.089 | |  |  |  |
| 25 – 0 | 66.7% | 48.2% | 82.8% | | | Beta (µ,σ) | µ=0.667, σ=0.089 | |  |  |  |
| Probability of cataract surgery for phakic with cataract patients | | | | | | | | | | |  |
| Prob of cataract surgery change of intercept | -0.17 | -0.96 | 0.63 | | | Normal+ (µ,σ) | µ=-0.169, σ=0.405 | | BC: logit model on FAME data  PSA, DSA: normal distribution assumed for intercept, 95% CI | |  |
| Prob of cataract surgery change of treatment eff | -0.21 | -0.95 | 0.53 | | | Normal+ (µ,σ) | µ=-0.212, σ=0.377 | |  |  |  |
| Mortality | | | | | | | | | | |  |
| RR of mortality (diabetes) | 1.95 | 1.64 | 2.33 | | | LogNormalY (µ,σ) | µ=0.670, σ=0.090 | | Preis et al. – 2009 | |  |
| RR of mortality (DMO) | 1.23 | 1.16 | 1.31 | | | LogNormalY (µ,σ) | µ=0.209, σ=0.031 | | Christ et al. - 2008, Ranibizumab Technology Appraisal Guidance 2013 Section 13.4, page 13. | |  |
| Adverse events | | | | | | | | | | |  |
| FAc - year 1 | | | | | | | | | | |  |
| Prob of IOP per cycle | 26.2% | 20.5% | 32.5% | | | Beta (µ,σ) | µ=0.263, σ=0.031 | | Previous Alimera model based on the FAME study [[20](#_ENREF_20)] | |  |
| Prob of retinal detachment | 1.0% | 0.6% | 1.6% | | | Beta (µ,σ) | µ=0.010, σ=0.003 | |  |  |  |
| Prob of endophthalmitis | 0.0% | 0.0% | 0.5% | | | Beta (µ,σ) | µ=0.002, σ=0.050 | |  |  |  |
| Prob of haemorrhage | 0.0% | 0.1% | 2.7% | | | Beta (µ,σ) | µ=0.010, σ=0.006 | |  |  |  |
| Prob of glaucoma | 0.0% | 0.0% | 1.8% | | | Beta (µ,σ) | µ=0.005, σ=0.050 | |  |  |  |
| FAc - year 2 | | | | | | | | | | |  |
| Prob of IOP per cycle | 13.6% | 9.1% | 18.8% | | | Beta (µ,σ) | µ=0.136, σ=0.025 | | Previous Alimera model | |  |
| Prob of retinal detachment | 1.0% | 0.6% | 1.6% | | | Beta (µ,σ) | µ=0.010, σ=0.003 | |  |  |  |
| Prob of endophthalmitis | 0.00% | 0.0% | 0.5% | | | Beta (µ,σ) | µ=0.002, σ=0.050 | |  |  |  |
| Prob of haemorrhage | 1.0% | 0.1% | 2.9% | | | Beta (µ,σ) | µ=0.010, σ=0.006 | |  |  |  |
| Prob of glaucoma | 2.1% | 0.6% | 4.5% | | | Beta (µ,σ) | µ=0.021, σ=0.011 | |  |  |  |
| FAc - year 3 | | | | | | | | | | |  |
| Prob of IOP per cycle | 9.6% | 5.7% | 14.4% | | | Beta (µ,σ) | µ=0.096, σ=0.022 | | Previous Alimera model | |  |
| Prob of retinal detachment | 1.0% | 0.6% | 1.6% | | | Beta (µ,σ) | µ=0.010, σ=0.003 | |  |  |  |
| Prob of endophthalmitis | 0.0% | 0.0% | 0.5% | | | Beta (µ,σ) | µ=0.002, σ=0.050 | |  |  |  |
| Prob of haemorrhage | 2.3% | 0.6% | 4.9% | | | Beta (µ,σ) | µ=0.023, σ=0.012 | |  |  |  |
| Prob of glaucoma | 5.7% | 2.8% | 9.5% | | | Beta (µ,σ) | µ=0.057, σ=0.018 | |  |  |  |
| Odds ratios (versus FAc) of occurrence of glaucoma | | | | | | | | | | |  |
| Bev or Ran | 0.618 | 0.01 | 3.37 | | | LogNormalY (µ,σ) | µ=-1.807, σ=1.542 | | Network meta-analysis, DSA: 95% CI | |  |
| Adverse events | | | | | | | | | | |  |
| Laser Photocoagulation | | | | | | | | | | |  |
| Prob of IOP per cycle | 5.3% | 3.1% | 8.1% | | | Beta (µ,σ) | µ=0.053, σ=0.013 | | Previous Alimera model | |  |
| Prob of retinal detachment | 1.0% | 0.6% | 1.6% | | | Beta (µ,σ) | µ=0.010, σ=0.003 | |  |  |  |
| Prob of endophthalmitis | 0.0% | 0.0% | 0.5% | | | Beta (µ,σ) | µ=0.002, σ=0.050 | |  |  |  |
| Prob of haemorrhage | 2.6% | 1.1% | 4.7% | | | Beta (µ,σ) | µ=0.026, σ=0.010 | |  |  |  |
| Prob of glaucoma | 0.0% | 0.0% | 0.0% | | | Beta (µ,σ) | µ=0.000, σ=0.000 | |  |  |  |
| Dexamethasone | | | | | | | | | | |  |
| Prob of IOP per cycle | 43.7% | 0.15 | 1.08 | | | LogNormalY (µ,σ) | µ=-0.917, σ=0.506 | | Adjusted indirect comparison | |  |
| Prob of retinal detachment | 0.0% | 0.0% | 0.3% | | | Beta (µ,σ) | µ=0.001, σ=0.000 | | Table 26, ERG report of aflibercept NICE submission (p.78) | |  |
| Prob of endophthalmitis | 0.0% | 0.0% | 0.3% | | | Beta (µ,σ) | µ=0.001, σ=0.000 | |  |  |  |
| Prob of haemorrhage | 2.1% | 0.6% | 4.5% | | | Beta (µ,σ) | µ=0.021, σ=0.011 | |  |  |  |
| Prob of glaucoma | 0.0% | 0.0% | 0.0% | | | Beta (µ,σ) | µ=0.000, σ=0.000 | |  |  |  |
| Bevacizumab or Ranibizumab | | | | | | | | | | |  |
| Prob of IOP per cycle | 3.6% | 0.01 | 0.16 | | | LogNormalY (µ,σ) | µ=-3.357, σ=0.768 | | Adjusted indirect comparison, DSA 95% CI | |  |
| Prob of retinal detachment | 0.0% | 0.0% | 0.0% | | | Beta (µ,σ) | µ=0.000, σ=0.000 | | Table 26, ERG report of aflibercept NICE submission (p.78), DSA 95% CI | |  |
| Prob of endophthalmitis | 0.0% | 0.0% | 0.3% | | | Beta (µ,σ) | µ=0.010, σ=0.000 | |  |  |  |
| Prob of haemorrhage | 0.0% | 0.0% | 0.0% | | | Beta (µ,σ) | µ=0.000, σ=0.000 | |  |  |  |
| Aflibercept | | | | | | | | | | |  |
| Prob of IOP per cycle | 3.6% | 0.01 | 0.16 | | | Beta (µ,σ) | µ=-3.357, σ=0.768 | | Adjusted indirect comparison, DSA 95% CI | |  |
| Prob of retinal detachment | 0.0% | 0.0% | 0.3% | | | Beta (µ,σ) | µ=0.001, σ=0.000 | | Table 26, ERG report of aflibercept NICE submission (p.78), DSA 95% CI | |  |
| Prob of endophthalmitis | 0.0% | 0.0% | 0.0% | | | Beta (µ,σ) | µ=0.000, σ=0.000 | |  |  |  |
| Prob of haemorrhage | 1.3% | 0.2% | 3.3% | | | Beta (µ,σ) | µ=0.013, σ=0.007 | |  |  |  |
| Prob of glaucoma | 3.4% | 1.4% | 6.3% | | | Beta (µ,σ) | µ=0.034, σ=0.013 | |  |  |  |
| Transition Matrices | | | | | | | | | | |  |
| Pseudophakic Population | | | | | | | | | | |  |
| FAc - 2 coefficient | -0.38 | -1.60 | 0.84 | | | Normal (µ,σ) | µ=-0.380, σ=0.622 | | Multinomial logistic model applied on patient’s level data from the ICE-UK, DSA: 95% CI | |  |
| FAc -1 coefficient | -0.30 | -1.11 | 0.52 | | | Normal (µ,σ) | µ=-0.295, σ=0.416 | |  |  |  |
| FAc 0 coefficient | 0.04 | -0.76 | 0.83 | | | Normal (µ,σ) | µ=0.373, σ=0.406 | |  |  |  |
| FAc +1 coefficient | 0.41 | -0.42 | 1.23 | | | Normal (µ,σ) | µ=0.408, σ=0.422 | |  |  |  |
| Full Population | | | | | | | | | | |  |
| FAc - 2 coefficient | 0.04 | -0.87 | 0.95 | | | Normal (µ,σ) | µ=0.040, σ=0.465 | | Multinomial logistic model applied on patient’s level data from the ICE-UK, DSA: 95% CI | |  |
| FAc -1 coefficient | -0.10 | -0.79 | 0.59 | | | Normal (µ,σ) | µ=-0.097, σ=0.351 | |  |  |  |
| FAc 0 coefficient | 0.22 | -0.42 | 0.85 | | | Normal (µ,σ) | µ=0.216, σ=0.323 | |  |  |  |
| FAc +1 coefficient | 0.51 | -0.14 | 1.16 | | | Normal (µ,σ) | µ=0.508, σ=0.333 | |  |  |  |
| Odds Ratios (versus laser-sham) for transition matrices | | | | | | | | | | |  |
| Dexamethasone – OR for TP | 0.90 | 0.60 | 1.43 | | | LogNormalY (µ,σ) | µ=-0.077, σ=0.222 | | Calibration, based on the NMA for mean change in BCVA from baseline to 24 months, DSA: 95%CI | |  |
| Anti-VEGFs – OR for TP | 2.36 | 2.15 | 2.60 | | | LogNormalY (µ,σ) | µ=0.861, σ=0.049 | |  |  |  |
| Natural decrease in BCVA for DMO patients | | | | | | | | | | |  |
| Probability of decline to next BCVA category | 3.5% | 2.3% | 5.0% | | | Beta (µ,σ) | µ=0.035, σ=0.007 | | Aflibercept NICE STA | |  |
| Costs | | | | | | | | | | |  |
| Drug | | | | | | | | | | |  |
| Iluvien | 4500 | 3400 | 5500 | | | - | - | | Alimera | |  |
| Laser photocoagulation | 0 | 0 | - | | | - | - | | No drug costs | |  |
| Usual Care | 367 | 220 | - | | | - | - | | BNF (January 2017) for aflibercept and dexamethasone, dm+d list price for ranibizumab  DSA min – 40% discount  No DSA max | |  |
| Dexamethasone (Ozurdex) | 870 | 522 | - | | | - | - | | BNF (January 2017); DSA min – 40% discount; No DSA max | |  |
| Number of drug administration per treatment | | | | | | | | | | |  |
| Iluvien | 1.00 |  |  |  |  | | |  | |  | |
| Laser | 0.98 | 0.74 | 1.23 | | | Normal (µ,σ) | µ=0.980, σ=0.112 | | ERG report of aflibercept Table 34, DSA: ±25% | |  |
| Usual Care | 1.68 | 1.31 | 2.05 | | | Normal (µ,σ) | µ=1.681, σ=0.189 | | ICE-UK, DSA: 95% CI | |  |
| Costs of adverse events management | | | | | | | | | | |  |
| Cataract surgery (per procedure) | 1687 | 1090 | 1753 | | | Normal+ (µ,σ) | µ=1687.208, σ=490.988 | | 'National Schedule of Reference Costs 2015/16 weighted average of  - BZ34A: Phacoemulsification Cataract Extraction and Lens Implant, with CC Score 4+: Elective inpatient  - BZ34B: Phacoemulsification Cataract Extraction and Lens Implant, with CC Score 2-3: Elective inpatient  - BZ34C: Phacoemulsification Cataract Extraction and Lens Implant, with CC Score 0-1: Elective inpatient  + 3 outpatient consultant-led visits  Calculation from NHS national schedule of reference costs for 2015/2016 lower/upper quartiles | |  |
| Elevated IOP (medication) | 781.58 | 586.2 | 976.98 | | | Normal+ (µ,σ) | µ=781.584, σ=289.476 | | BNF (January 2017) and assuming  - Equal weighting between Timolol, Xalatan, Azopt, Cosopt and Brimonidine tartrate  - Patients who have raised IOP will receive up to 3 years of treatment, consistent with the assumed duration of treatment for DMO  - Maximum of day per bottle of 28 days (maximum shelf-life per bottle)  + 6 extra IOP check  DSA - Assumed +/- 25% | |  |
| Elevated IOP (surgery) | 1156.2 | 813.3 | 1485.1 | | | Normal+ (µ,σ) | µ=1156.201, σ=497.636 | | 'National Schedule of Reference Costs 2015/16 : BZ87A - minor vitreous retinal procedure, 19 years and over. Non-elective short stay  + 4 outpatient visits  Calculation from NHS national schedule of reference costs for 2015/2016 lower/upper quartiles | |  |
| Retinal detachment repair (per procedure) | 1078.3 | 561.8 | 1415.0 | | | Normal+ (µ,σ) | µ=1078.313, σ=632.004 | | 'National Schedule of Reference Costs 2015/16 : BZ87A - minor vitreous retinal procedure, 19 years and over. Non-elective short stay  + 4 outpatient visits  Calculation from NHS national schedule of reference costs for 2015/2016 lower/upper quartiles | |  |
| Endophthalmitis (per case) | 1186.9 | 776.3 | 1406.2 | | | Normal+ (µ,σ) | µ=1186.944, σ=466.630 | | 'National Schedule of Reference Costs 2015/16: BZ86B - Intermediate Vitreous Retinal Procedures, 19 years and over, with CC Score 0-1 Non-elective long stay  + 6 outpatient consultant-led visits  Calculation from NHS national schedule of reference costs for 2015/2016 lower/upper quartiles | |  |
| Vitrectomy | 841.75 | 369.0 | 1129.0 | | | Normal+ (µ,σ) | µ=841.753, σ=562.963 | | 'National Schedule of Reference Costs 2015/16 - BZ87A - minor vitreous retinal procedure, 19 years and over: Non-elective short stay -  + 2 outpatient consultant-led visits  Calculation from NHS national schedule of reference costs for 2015/2016 lower/upper quartiles | |  |
| Glaucoma | 1156.2 | 813.3 | 1485.1 | | | Normal+ (µ,σ) | µ=1156.201, σ=497.636 | | Assumed as Dexamethasone ERG:  - 50% National Schedule of Reference Costs Year : 2015- 16 -BZ94B - Intermediate, Glaucoma or Iris Procedures, with CC Score 0  - 50% National Schedule of Reference Costs Year : 2015 - 16 -BZ93B - Major, Glaucoma or Iris Procedures, with CC Score 0-1  + 6 extra IOP check  Calculation from NHS national schedule of reference costs for 2015/2016 lower/upper quartiles | |  |
| Blindness | 3002 | 2251 | 3377 | | | Normal+ (µ,σ) | µ=3001.510, σ=833.753 | | Updated values from Meads et al. | |  |
| Outpatient visits - year 1 | | | | | | | | | | |  |
| FAc - year 1 | 5.60 | 3.40 | 7.80 | | | Normal (µ,σ) | µ=5.600, σ=1.120 | | ICE-UK, DSA: 95% CI | |  |
| Laser -year 1 | 4.00 | 2.43 | 5.57 | | | Normal (µ,σ) | µ=4.000, σ=0.800 | | Aflibercept NICE STA, DSA: 95% CI | |  |
| Ranibizumab - year 1 | 12.00 | 7.30 | 16.70 | | | Normal (µ,σ) | µ=12.000, σ=2.400 | | Aflibercept NICE STA, DSA: 95% CI | |  |
| Bevacizumab - year 1 | 12.00 | 7.30 | 16.70 | | | Normal (µ,σ) | µ=12.000, σ=2.400 | | Assumption (equal to ranibizumab) | |  |
| Aflibercept - year 1 | 8.00 | 4.86 | 11.14 | | | Normal (µ,σ) | µ=8.000, σ=1.600 | | Aflibercept NICE STA, DSA: 95% CI | |  |
| Dexamethasone - year 1 | 4.53 | 2.76 | 6.31 | | | Normal (µ,σ) | µ=4.530, σ=0.907 | | Maximum from dexamethasone NICE STA data and ICE-UK, Average for 3 years, DSA: 95% CI | |  |
| Outpatient visits - year 2 | | | | | | | | | | |  |
| FAc - year 2 | 2.80 | 1.70 | 3.90 | | | Normal (µ,σ) | µ=2.800, σ=0.560 | | ICE-UK DSA: 95% CI | |  |
| Laser -year 2 | 4.00 | 2.43 | 5.57 | | | Normal (µ,σ) | µ=4.000, σ=0.800 | | Aflibercept NICE STA DSA: 95% CI | |  |
| Ranibizumab - year 2 | 6.30 | 3.83 | 8.77 | | | Normal (µ,σ) | µ=6.300, σ=1.260 | | Aflibercept NICE STA DSA: 95% CI | |  |
| Bevacizumab - year 2 | 6.30 | 3.83 | 8.77 | | | Normal (µ,σ) | µ=6.300, σ=1.260 | | Assumption (equal to ranibizumab) | |  |
| Aflibercept - year 2 | 6.30 | 3.83 | 8.77 | | | Normal (µ,σ) | µ=6.300, σ=1.260 | | Aflibercept NICE STA DSA: 95% CI | |  |
| Dexamethasone - year 2 | 4.53 | 2.76 | 6.31 | | | Normal (µ,σ) | µ=4.533, σ=0.907 | | Maximum from dexamethasone NICE STA data and ICE-UK, Average for 3 years DSA: 95% CI | |  |
| Outpatient visits - year 3 | | | | | | | | | | |  |
| FAc - year 3 | 3.00 | 1.82 | 4.18 | | | Normal (µ,σ) | µ=3.000, σ=0.600 | | ICE-UK DSA: 95% CI | |  |
| Laser -year 3 | 2.60 | 1.58 | 3.62 | | | Normal (µ,σ) | µ=2.600, σ=0.520 | | Aflibercept NICE STA DSA: 95% CI | |  |
| Ranibizumab - year 3 | 4.00 | 2.43 | 5.57 | | | Normal (µ,σ) | µ=4.000, σ=0.800 | | Aflibercept NICE STA DSA: 95% CI | |  |
| Bevacizumab - year 3 | 4.00 | 2.43 | 5.57 | | | Normal (µ,σ) | µ=4.000, σ=0.800 | | Assumption (equal to ranibizumab) | |  |
| Aflibercept - year 3 | 4.00 | 2.43 | 5.57 | | | Normal (µ,σ) | µ=4.000, σ=0.800 | | Aflibercept NICE STA DSA: 95% CI | |  |
| Dexamethasone - year 3 | 4.53 | 2.76 | 6.31 | | | Normal (µ,σ) | µ=4.533, σ=0.907 | | Maximum from dexamethasone NICE STA data and ICE-UK, Average for 3 years DSA: 95% CI | |  |
| Optical coherence tomography - year 1 | | | | | | | | | | |  |
| FAc - year 1 | 3.30 | 2.01 | 4.59 | | | Normal (µ,σ) | µ=3.300, σ=0.660 | | ICE-UK DSA: 95% CI | |  |
| Laser -year 1 | 4.00 | 2.43 | 5.57 | | | Normal (µ,σ) | µ=4.000, σ=0.800 | | Aflibercept NICE STA DSA: 95% CI | |  |
| Ranibizumab - year 1 | 12.00 | 7.30 | 16.70 | | | Normal (µ,σ) | µ=12.000, σ=2.400 | | Aflibercept NICE STA DSA: 95% CI | |  |
| Bevacizumab - year 1 | 12.00 | 7.30 | 16.70 | | | Normal (µ,σ) | µ=12.000, σ=2.400 | | Assumption (equal to ranibizumab) | |  |
| Aflibercept - year 1 | 8.00 | 4.86 | 11.14 | | | Normal (µ,σ) | µ=8.000, σ=1.600 | | Aflibercept NICE STA DSA: 95% C DSA: 95% CI I | |  |
| Dexamethasone - year 1 | 4.00 | 2.43 | 5.57 | | | Normal (µ,σ) | µ=4.000, σ=0.800 | | Maximum from dexamethasone NICE STA data and ICE-UK, Average for 3 years | |  |
| Optical coherence tomography - year 2 | | | | | | | | | | |  |
| FAc - year 2 | 1.70 | 1.03 | 2.37 | | | Normal (µ,σ) | µ=1.700, σ=0.340 | | ICE-UK DSA: 95% CI | |  |
| Laser -year 2 | 4.00 | 2.43 | 5.57 | | | Normal (µ,σ) | µ=4.000, σ=0.800 | | Aflibercept NICE STA DSA: 95% CI | |  |
| Ranibizumab - year 2 | 6.30 | 3.83 | 8.77 | | | Normal (µ,σ) | µ=6.300, σ=1.260 | | Aflibercept NICE STA DSA: 95% CI | |  |
| Bevacizumab - year 2 | 6.30 | 3.83 | 8.77 | | | Normal (µ,σ) | µ=6.300, σ=1.260 | | Assumption (equal to ranibizumab) | |  |
| Aflibercept - year 2 | 6.30 | 3.83 | 8.77 | | | Normal (µ,σ) | µ=6.300, σ=1.260 | | Aflibercept NICE STA DSA: 95% CI | |  |
| Dexamethasone - year 2 | 4.00 | 2.43 | 5.57 | | | Normal (µ,σ) | µ=4.000, σ=0.800 | | Maximum from dexamethasone NICE STA data and ICE-UK, Average for 3 years DSA: 95% CI | |  |
| Optical coherence tomography - year 3 | | | | | | | | | | |  |
| FAc - year 3 | 3.00 | 1.82 | 4.18 | | | Normal (µ,σ) | µ=3.000, σ=0.600 | | ICE-UK DSA: 95% CI | |  |
| Laser -year 3 | 2.60 | 1.58 | 3.62 | | | Normal (µ,σ) | µ=2.600, σ=0.520 | | Aflibercept NICE STA DSA: 95% CI | |  |
| Ranibizumab - year 3 | 4.00 | 2.43 | 5.57 | | | Normal (µ,σ) | µ=4.000, σ=0.800 | | Aflibercept NICE STA DSA: 95% CI | |  |
| Bevacizumab - year 3 | 4.00 | 2.43 | 5.57 | | | Normal (µ,σ) | µ=4.000, σ=0.800 | | Assumption (equal to ranibizumab) | |  |
| Aflibercept - year 3 | 4.00 | 2.43 | 5.57 | | | Normal (µ,σ) | µ=4.000, σ=0.800 | | Aflibercept NICE STA DSA: 95% CI | |  |
| Dexamethasone - year 3 | 4.00 | 2.43 | 5.57 | | | Normal (µ,σ) | µ=4.000, σ=0.800 | | Maximum from dexamethasone NICE STA data and ICE-UK, Average for 3 years DSA: 95% CI | |  |
| Fluorescence angiography - year 1 | | | | | | | | | | |  |
| FAc - year 1 | 1.00 | 0.61 | 1.39 | | | Normal (µ,σ) | µ=1.000, σ=0.200 | | ICE-UK DSA: 95% CI | |  |
| Laser -year 1 | 1.00 | 0.61 | 1.39 | | | Normal (µ,σ) | µ=1.000, σ=0.200 | | Aflibercept NICE STA DSA: 95% CI | |  |
| Ranibizumab - year 1 | 1.00 | 0.61 | 1.39 | | | Normal (µ,σ) | µ=1.000, σ=0.200 | | Aflibercept NICE STA DSA: 95% CI | |  |
| Bevacizumab - year 1 | 1.00 | 0.61 | 1.39 | | | Normal (µ,σ) | µ=1.000, σ=0.200 | | Assumption (equal to ranibizumab) | |  |
| Aflibercept - year 1 | 1.00 | 0.61 | 1.39 | | | Normal (µ,σ) | µ=1.000, σ=0.200 | | Aflibercept NICE STA DSA: 95% CI | |  |
| Dexamethasone - year 1 | 1.00 | 0.61 | 1.39 | | | Normal (µ,σ) | µ=1.000, σ=0.200 | | Maximum from dexamethasone NICE STA data and ICE-UK, Average for 3 years DSA: 95% CI | |  |
| Fluorescence angiography - year 2 | | | | | | | | | | |  |
| FAc - year 2 | 1.00 | 0.61 | 1.39 | | | Normal (µ,σ) | µ=1.000, σ=0.200 | | ICE-UK DSA: 95% CI | |  |
| Laser -year 2 | 0.00 | 0.00 | 0.00 | | | Normal (µ,σ) | µ=0.000, σ=0.000 | | Aflibercept NICE STA DSA: 95% CI | |  |
| Ranibizumab - year 2 | 0.00 | 0.00 | 0.00 | | | Normal (µ,σ) | µ=0.000, σ=0.000 | | Aflibercept NICE STA DSA: 95% CI | |  |
| Bevacizumab - year 2 | 0.00 | 0.00 | 0.00 | | | Normal (µ,σ) | µ=0.000, σ=0.000 | | Assumption (equal to ranibizumab) | |  |
| Aflibercept - year 2 | 0.00 | 0.00 | 0.00 | | | Normal (µ,σ) | µ=0.000, σ=0.000 | | Aflibercept NICE STA DSA: 95% CI | |  |
| Dexamethasone - year 2 | 1.00 | 0.61 | 1.39 | | | Normal (µ,σ) | µ=1.000, σ=0.200 | | Maximum from dexamethasone NICE STA data and ICE-UK, Average for 3 years DSA: 95% CI | |  |
| Fluorescence angiography - year 3 | | | | | | | | | | |  |
| FAc - year 3 | 1.00 | 0.61 | 1.39 | | | Normal (µ,σ) | µ=1.000, σ=0.200 | | ICE-UK DSA: 95% CI | |  |
| Laser -year 3 | 0.00 | 0.00 | 0.00 | | | Normal (µ,σ) | µ=0.000, σ=0.000 | | Aflibercept NICE STA DSA: 95% CI | |  |
| Ranibizumab - year 3 | 0.00 | 0.00 | 0.00 | | | Normal (µ,σ) | µ=0.000, σ=0.000 | | Aflibercept NICE STA DSA: 95% CI | |  |
| Bevacizumab - year 3 | 0.00 | 0.00 | 0.00 | | | Normal (µ,σ) | µ=0.000, σ=0.000 | | Assumption (equal to ranibizumab) | |  |
| Aflibercept - year 3 | 0.00 | 0.00 | 0.00 | | | Normal (µ,σ) | µ=0.000, σ=0.000 | | Aflibercept NICE STA DSA: 95% CI | |  |
| Dexamethasone - year 3 | 1.00 | 0.61 | 1.39 | | | Normal (µ,σ) | µ=1.000, σ=0.200 | | Maximum from dexamethasone NICE STA data and ICE-UK, Average for 3 years DSA: 95% CI | |  |
| Intraocular pressure check - year 1 | | | | | | | | | | |  |
| FAc - year 1 | 3.60 | 2.19 | 5.01 | | | Normal (µ,σ) | µ=3.600, σ=0.720 | | ICE-UK DSA: 95% CI | |  |
| Laser -year 1 | 4.00 | 2.43 | 5.57 | | | Normal (µ,σ) | µ=4.000, σ=0.800 | | Aflibercept NICE STA DSA: 95% CI | |  |
| Ranibizumab - year 1 | 12.00 | 7.30 | 16.70 | | | Normal (µ,σ) | µ=12.000, σ=2.400 | | Aflibercept NICE STA DSA: 95% CI | |  |
| Bevacizumab - year 1 | 12.00 | 7.30 | 16.70 | | | Normal (µ,σ) | µ=12.000, σ=2.400 | | Assumption (equal to ranibizumab) | |  |
| Aflibercept - year 1 | 8.00 | 4.86 | 11.14 | | | Normal (µ,σ) | µ=8.000, σ=1.600 | | Aflibercept NICE STA DSA: 95% CI | |  |
| Dexamethasone - year 1 | 2.53 | 1.54 | 3.53 | | | Normal (µ,σ) | µ=2.533, σ=0.507 | | Maximum from dexamethasone NICE STA data and ICE-UK, Average for 3 years DSA: 95% CI | |  |
| Intraocular pressure check - year 2 | | | | | | | | | | |  |
| FAc - year 2 | 1.90 | 1.16 | 2.64 | | | Normal (µ,σ) | µ=1.900, σ=0.380 | | ICE-UK DSA: 95% CI | |  |
| Laser -year 2 | 4.00 | 2.43 | 5.57 | | | Normal (µ,σ) | µ=4.000, σ=0.800 | | Aflibercept NICE STA DSA: 95% CI | |  |
| Ranibizumab - year 2 | 6.30 | 3.83 | 8.77 | | | Normal (µ,σ) | µ=6.300, σ=1.260 | | Aflibercept NICE STA DSA: 95% CI | |  |
| Bevacizumab - year 2 | 6.30 | 3.83 | 8.77 | | | Normal (µ,σ) | µ=6.300, σ=1.260 | | Assumption (equal to ranibizumab) | |  |
| Aflibercept - year 2 | 6.30 | 3.83 | 8.77 | | | Normal (µ,σ) | µ=6.300, σ=1.260 | | Aflibercept NICE STA DSA: 95% CI | |  |
| Dexamethasone - year 2 | 2.53 | 1.54 | 3.53 | | | Normal (µ,σ) | µ=2.533, σ=0.507 | | Maximum from dexamethasone NICE STA data and ICE-UK, Average for 3 years DSA: 95% CI | |  |
| Intraocular pressure check - year 3 | | | | | | | | | | |  |
| FAc - year 3 | 0.00 | 0.00 | 0.00 | | | Normal (µ,σ) | µ=1.900, σ=0.380 | | ICE-UK DSA: 95% CI | |  |
| Laser -year 3 | 2.60 | 1.58 | 3.62 | | | Normal (µ,σ) | µ=2.600, σ=0.520 | | Aflibercept NICE STA DSA: 95% CI | |  |
| Ranibizumab - year 3 | 4.00 | 2.43 | 5.57 | | | Normal (µ,σ) | µ=4.000, σ=0.800 | | Aflibercept NICE STA DSA: 95% CI | |  |
| Bevacizumab - year 3 | 4.00 | 2.43 | 5.57 | | | Normal (µ,σ) | µ=4.000, σ=0.800 | | Assumption (equal to ranibizumab) | |  |
| Aflibercept - year 3 | 4.00 | 2.43 | 5.57 | | | Normal (µ,σ) | µ=4.000, σ=0.800 | | Aflibercept NICE STA DSA: 95% CI | |  |
| Dexamethasone - year 3 | 2.53 | 1.54 | 3.53 | | | Normal (µ,σ) | µ=2.533, σ=0.507 | | Maximum from dexamethasone NICE STA data and ICE-UK, Average for 3 years DSA: 95% CI | |  |
| Utilities per health state | | | | | | | | | | |  |
| Utility decrements | | | | | | | | | | |  |
| BSE - 86-100, WSE - 86-100 | 0.000 | -0.034 | 0.034 | | | Normal (µ,σ) | µ=0.000, σ=0.017 | | BC: Czoski-Murray et al. 2009  DSA: For each utility value the maximum difference was calculated based on the two alternative data sources (Brown et al (1999), Brown et al (2000)) in comparison to the base case  PSA: Normal distribution was assumed based on the range from DSA understand as 95% CI | |  |
| BSE - 86-100, WSE - 76-85 | 0.000 | -0.055 | 0.055 | | | Normal (µ,σ) | µ=0.000, σ=0.028 | |  |  |  |
| BSE - 86-100, WSE - 66-75 | 0.000 | -0.054 | 0.054 | | | Normal (µ,σ) | µ=0.000, σ=0.027 | |  |  |  |
| BSE - 86-100, WSE - 56-65 | 0.000 | -0.043 | 0.043 | | | Normal (µ,σ) | µ=0.000, σ=0.022 | |  |  |  |
| BSE - 86-100, WSE - 46-55 | 0.000 | -0.031 | 0.031 | | | Normal (µ,σ) | µ=0.000, σ=0.016 | |  |  |  |
| BSE - 86-100, WSE - 36-45 | 0.000 | -0.044 | 0.044 | | | Normal (µ,σ) | µ=0.000, σ=0.022 | |  |  |  |
| BSE - 86-100, WSE - 26-35 | 0.000 | -0.055 | 0.055 | | | Normal (µ,σ) | µ=0.000, σ=0.028 | |  |  |  |
| BSE - 86-100, WSE - 0-25 | 0.000 | -0.072 | 0.072 | | | Normal (µ,σ) | µ=0.000, σ=0.037 | |  |  |  |
| BSE - 76-85, WSE - 76-85 | 0.000 | -0.126 | 0.126 | | | Normal (µ,σ) | µ=0.000, σ=0.064 | |  |  |  |
| BSE - 76-85, WSE - 66-75 | 0.000 | -0.125 | 0.125 | | | Normal (µ,σ) | µ=0.000, σ=0.064 | |  |  |  |
| BSE - 76-85, WSE - 56-65 | 0.000 | -0.114 | 0.114 | | | Normal (µ,σ) | µ=0.000, σ=0.058 | |  |  |  |
| BSE - 76-85, WSE - 46-55 | 0.000 | -0.101 | 0.101 | | | Normal (µ,σ) | µ=0.000, σ=0.052 | |  |  |  |
| BSE - 76-85, WSE - 36-45 | 0.000 | -0.115 | 0.115 | | | Normal (µ,σ) | µ=0.000, σ=0.058 | |  |  |  |
| BSE - 76-85, WSE - 26-35 | 0.000 | -0.126 | 0.126 | | | Normal (µ,σ) | µ=0.000, σ=0.064 | |  |  |  |
| BSE - 76-85, WSE - 0-25 | 0.000 | -0.142 | 0.142 | | | Normal (µ,σ) | µ=0.000, σ=0.073 | |  |  |  |
| BSE - 66-75, WSE - 66-75 | 0.000 | -0.120 | 0.120 | | | Normal (µ,σ) | µ=0.000, σ=0.061 | |  |  |  |
| BSE - 66-75, WSE - 56-65 | 0.000 | -0.110 | 0.110 | | | Normal (µ,σ) | µ=0.000, σ=0.056 | |  |  |  |
| BSE - 66-75, WSE - 46-55 | 0.000 | -0.115 | 0.115 | | | Normal (µ,σ) | µ=0.000, σ=0.059 | |  |  |  |
| BSE - 66-75, WSE - 36-45 | 0.000 | -0.120 | 0.120 | | | Normal (µ,σ) | µ=0.000, σ=0.061 | |  |  |  |
| BSE - 66-75, WSE - 26-35 | 0.000 | -0.125 | 0.125 | | | Normal (µ,σ) | µ=0.000, σ=0.064 | |  |  |  |
| BSE - 66-75, WSE - 0-25 | 0.000 | -0.144 | 0.144 | | | Normal (µ,σ) | µ=0.000, σ=0.074 | |  |  |  |
| BSE - 56-65, WSE - 56-65 | 0.000 | -0.166 | 0.166 | | | Normal (µ,σ) | µ=0.000, σ=0.085 | |  |  |  |
| BSE - 56-65, WSE - 46-55 | 0.000 | -0.172 | 0.172 | | | Normal (µ,σ) | µ=0.000, σ=0.088 | |  |  |  |
| BSE - 56-65, WSE - 36-45 | 0.000 | -0.177 | 0.177 | | | Normal (µ,σ) | µ=0.000, σ=0.090 | |  |  |  |
| BSE - 56-65, WSE - 26-35 | 0.000 | -0.182 | 0.182 | | | Normal (µ,σ) | µ=0.000, σ=0.093 | |  |  |  |
| BSE - 56-65, WSE - 0-25 | 0.000 | -0.201 | 0.201 | | | Normal (µ,σ) | µ=0.000, σ=0.102 | |  |  |  |
| BSE - 46-55, WSE - 46-55 | 0.000 | -0.189 | 0.189 | | | Normal (µ,σ) | µ=0.000, σ=0.096 | |  |  |  |
| BSE - 46-55, WSE - 36-45 | 0.000 | -0.194 | 0.194 | | | Normal (µ,σ) | µ=0.000, σ=0.099 | |  |  |  |
| BSE - 46-55, WSE - 26-35 | 0.000 | -0.199 | 0.199 | | | Normal (µ,σ) | µ=0.000, σ=0.102 | |  |  |  |
| BSE - 46-55, WSE - 0-25 | 0.000 | -0.218 | 0.218 | | | Normal (µ,σ) | µ=0.000, σ=0.111 | |  |  |  |
| BSE - 36-45, WSE - 36-45 | 0.000 | -0.212 | 0.212 | | | Normal (µ,σ) | µ=0.000, σ=0.108 | |  |  |  |
| BSE - 36-45, WSE - 26-35 | 0.000 | -0.217 | 0.217 | | | Normal (µ,σ) | µ=0.000, σ=0.111 | |  |  |  |
| BSE - 36-45, WSE - 0-25 | 0.000 | -0.236 | 0.236 | | | Normal (µ,σ) | µ=0.000, σ=0.120 | |  |  |  |
| BSE - 26-35, WSE - 26-35 | 0.000 | -0.234 | 0.234 | | | Normal (µ,σ) | µ=0.000, σ=0.119 | |  |  |  |
| BSE - 26-35, WSE - 0-25 | 0.000 | -0.253 | 0.253 | | | Normal (µ,σ) | µ=0.000, σ=0.129 | |  |  |  |
| BSE - 0-25, WSE - 0-25 | 0.000 | -0.316 | 0.316 | | | Normal (µ,σ) | µ=0.000, σ=0.161 | |  |  |  |
| Utility decrements | | | | | | | | | | |  |
| IOP | 0.000 | 0.000 | 0.000 | | | Normal (µ,σ) | µ=0.000, σ=0.000 | | Evidence Review Group report for aflibercept submission (p. 82)  DSA: 95% CI | |  |
| Retinal detachment repair | 0.130 | 0.079 | 0.181 | | | Normal (µ,σ) | µ=0.130, σ=0.026 | |  |  |  |
| Endophthalmitis | 0.000 | 0.000 | 0.000 | | | Normal (µ,σ) | µ=0.000, σ=0.000 | |  |  |  |
| Vitreous haemorrhage | 0.020 | 0.012 | 0.028 | | | Normal (µ,σ) | µ=0.020, σ=0.004 | |  |  |  |
| Glaucoma procedure | 0.000 | 0.000 | 0.000 | | | Normal (µ,σ) | µ=0.000, σ=0.000 | |  |  |  |
| Injection therapy (anxiety) | 0.071 | 0.043 | 0.099 | | | Normal (µ,σ) | µ=0.071, σ=0.014 | | UK population norms EQ-5D (<https://www.york.ac.uk/che/pdf/DP172.pdf>) DSA: 95% CI | |  |
| Percentage of patients with anxiety | 0.173 | 0.133 | 0.218 | | | Beta (µ,σ) | µ=0.173, σ=0.022 | | Senra et.al. 2017 DSA: 95% CI | |  |
